# Depletion of Boric Acid and Cobalt from Cultivation Media: Impact on Recombinant Protein Production with *Komagataella phaffii*

**DOI:** 10.3390/bioengineering7040161

**Published:** 2020-12-13

**Authors:** Alexander Pekarsky, Sophia Mihalyi, Maximilian Weiss, Andreas Limbeck, Oliver Spadiut

**Affiliations:** 1 Institute of Chemical, Environmental and Bioscience Engineering, TU Wien, Gumpendorferstrasse 1a, 1060 Vienna, Austria; alexander.pekarsky@tuwien.ac.at (A.P.); sophia.mihalyi@students.boku.ac.at (S.M.); 2 Institute of Chemical Technologies and Analytics, TU Wien, Getreidemarkt 9/164-I2AC, 1060 Vienna, Austria; maximilian.weiss@tuwien.ac.at (M.W.); andreas.limbeck@tuwien.ac.at (A.L.)

**Keywords:** *Pichia pastoris*, *Komagataella phaffii*, yeast, REACH-regulation, cobalt, boric acid, boron, protein quality

## Abstract

The REACH regulation stands for “Registration, Evaluation, Authorization and Restriction of Chemicals” and defines certain substances as harmful to human health and the environment. This urges manufacturers to adapt production processes. Boric acid and cobalt dichloride represent such harmful ingredients, but are commonly used in yeast cultivation media. The yeast *Komagataella phaffii* (*Pichia pastoris*) is an important host for heterologous protein production and compliance with the REACH regulation is desirable. Boric acid and cobalt dichloride are used as boron and cobalt sources, respectively. Boron and cobalt support growth and productivity and a number of cobalt-containing enzymes exist. Therefore, depletion of boric acid and cobalt dichloride could have various negative effects, but knowledge is currently scarce. Herein, we provide an insight into the impact of boric acid and cobalt depletion on recombinant protein production with *K. phaffii* and additionally show how different vessel materials affect cultivation media compositions through leaking elements. We found that boric acid could be substituted through boron leakiness from borosilicate glassware. Furthermore, depletion of boric acid and cobalt dichloride neither affected high cell density cultivation nor cell morphology and viability on methanol. However, final protein quality of three different industrially relevant enzymes was affected in various ways.

## 1. Introduction

Yeasts are currently used in numerous biotechnological production processes. Frequently used yeasts are *Saccharomyces cerevisiae*, *Komagataella phaffii* (*Pichia pastoris*), *Kluyveromyces lactis* and *Yarrowia lipolytica* [1]. *K. lactis* finds applications in the dairy industry and gains importance for recombinant protein production [2]. *Y. lipolytica* degrades hydrophobic substances, such as n-paraffins and oils, which highlights its usability in bioremediation [3]. The well-known budding yeasts *S. cerevisiae* and *K. phaffii* are used as eukaryotic model organisms, as hosts for recombinant protein production and in the case of *S. cerevisiae*, also for ethanol production [4,5,6]. Interestingly, both yeasts are also investigated in preclinical and clinical trials as whole-cell therapeutics [7]. Regarding recombinant protein production, *K. phaffii* shows lower hyper-mannosylation compared to *S. cerevisiae* [8], has strong promoters for growth on various C-sources from methanol to glucose [9] and a proven track record on glycoengineering to yield customized protein glycosylation [10].

Implemented in 2006 by the European Union, the REACH (Registration, Evaluation, Authorization and Restriction of Chemicals) regulation targets reduction of hazardous chemicals in manufacturing processes to protect human health and the environment [11]. Its implementation is meant to generate more data on safety and toxicity of substances and to avoid substances of high concern without approval [12]. However, during cultivation of yeasts, various substances are required to promote growth, metabolic activity and product formation.

The use of substances that contain lead, mercury, nickel or cadmium is restricted under REACH [13], but these elements are not needed for yeast cultivation. An important chemically defined medium for high cell density cultivation of *K. phaffii* is Basal Salt Medium (BSM), which contains salts, a phosphate source (H_3_PO_4_) and a nitrogen source (NH_4_OH) and is supplemented with a Pichia Trace Metal (PTM) solution that contains various metal salts, boric acid and biotin [14,15]. The historically important cultivation guideline “Pichia fermentation process guidelines” specifically mentions the use of defined BSM with PTM1 [16]. This defined cultivation medium is popular for high cell density cultivations (e.g., [17,18,19,20,21,22,23,24]), although other chemically defined media are known in the literature (e.g., [25,26,27,28]).

Importantly, all published chemically defined media for *K. phaffii* contain a PTM solution to support growth (e.g., [17,18,19,20,21,22,23,24,25,26,27,28]). This PTM solution usually contains boric acid as a boron source and cobalt dichloride as a cobalt source, which are classified as “substances of very high concern” by REACH. It is stated that boric acid may damage fertility or even the unborn child at concentrations ≥ 5.5% (*w/w*) [29]. The main concerns for cobalt and cobalt dichloride are carcinogenic effects, suspected mutagenicity, toxicity to reproduction, skin and respiratory sensitizing ≥0.01% (*w/w*) as well as toxicity to aquatic life ≥0.025% (*w/w*) [30,31]. The standard PTM1 solution contains 0.02 g·L^−1^ (~2 × 10^−3^% (*w/w*)) boric acid and 0.5 g·L^−1^ (~0.05% (*w/w*)) cobalt dichloride [15,16]. It must be mentioned that an average of 4.35 mL PTM1 solution is added to 1 L BSM and sometimes also to feed medium (e.g., [17,18,19,20,21,22,23,24,25,26,27,28]). This results in a final concentration of 0.09 mg·L^−1^ (~9 × 10^−6^% (*w/w*)) boric acid and 2.2 mg·L^−1^ (~2.2 × 10^−4^% (*w/w*)) cobalt dichloride, which marks a sufficient difference between used and allowed concentrations to comply with the REACH regulation. Nevertheless, the REACH regulation also mentions that one should aim to “progressively replace substances of very high concern with less dangerous substances” [32], which urges depletion of cobalt and boric acid from yeast cultivation media, if possible.

Unfortunately, there exists only scarce knowledge on cultivations without cobalt or boric acid. First, the boron metabolism in yeasts as well as in higher organisms is not fully understood yet [33,34]. Boron is an essential element for plants [35] and animals [36,37,38] and a number of boron transporters were identified in yeasts [39,40,41]. Some bacteria require boron for growth, nitrogen fixation and as a component of bactericides, like boromycin, tartrolon, borophisin or aplasmomycin [42]. Boron was found to stimulate growth of *S. cerevisiae* [43], but not of *K. phaffii* [44]. However, compared to the precise study with *S. cerevisiae* [43], the authors of the *K. phaffii* study did not account for potential boron contamination from other sources [44]. In higher eukaryotes, cobalt is mostly found in enzymes that contain the cyanocobalamin vitamin B12 (B12) [45,46]. However, yeasts do not require B12 [47] and hardly accumulate cobalt [48]. It was also shown that *K. phaffii* does not require cobalt for growth on glycerol and methanol, but that a PTM1 solution with cobalt boosted production of active β-galactosidase [44]. Importantly, cobalt transporters exist [49] and a limited number of cobalt metalloenzymes (methionine aminopeptidases) are currently known in yeasts [50,51]. Concluding, complete depletion of boric acid and cobalt from yeast cultivations might decrease growth and/or heterologous protein production, but more data are definitely needed.

It must be mentioned that substitution of boric acid and cobalt salts is tricky, because organic boron compounds raise environmental questions [52] and substitution of cobalt salts through B12 is hindered by the inability of yeasts to use B12 [47]. Interestingly, one approach to substitute the use of pure boric acid could be the use of borosilicate glassware that reportedly leaks boron over time [53,54].

In the current study, we investigated the impact of boric acid and cobalt dichloride depletion on high cell density cultivation, cellular morphology, viability and recombinant protein production with the yeast *K. phaffii*. Production and protein quality (i.e., kinetic variables, thermal stability and *N*-glycosylation) were investigated for three different, industrially relevant enzymes, namely the copper metalloenzyme galactose oxidase (GalOx), the heme-containing horseradish peroxidase (HRP) and a heme and FAD-containing flavocytochrome (FC). All experiments were performed under methanol induction, which is restricted to ≥3% (*w/w*) by REACH [55]. *K. phaffii* cultivations were either fed to yield methanol-limited growth or by methanol pulses to maximum concentrations of 0.5% (*w/w*). Our results show that high cell density cultivation is not limited by depletion of boric acid and cobalt, but that final recombinant protein quality is affected, which urges the need to investigate each target protein individually.

## 2. Materials and Methods

### 2.1. Chemicals and Reagents

Deionized water (MQ) was obtained from a Milli-Q system (Merck KGaA, Darmstadt, Germany).

D-Glucose monohydrate, D-Galactose, glycerol Solvagreen^®^ ≥98% Ph.Eur. anhydrous, 25% ammonia solution, yeast nitrogen base without amino acids/with ammonium sulfate (YNB), BisTRIS, TRIS, 30% hydrogen peroxide solution, potassium dihydrogen phosphate, calcium sulfate dihydrate, potassium sulfate, magnesium sulfate heptahydrate, 85% potassium hydroxide, anhydrous copper(II) sulfate ≥99%, sodium iodide, manganese sulfate monohydrate, zinc dichloride, sulfuric acid 98%, ammonium sulfate, calcium dichloride, potassium chloride, magnesium dichloride hexahydrate, sodium chloride, disodium hydrogen phosphate dihydrate and trisodium citrate trihydrate were obtained from Carl Roth (Karlsruhe, Germany).

Dipotassium hydrogen phosphate, sodium molybdate dihydrate and cobalt dichloride hexahydrate were obtained from Merck KGaA (Darmstadt, Germany).

D-Biotin, pure methanol, 85% ortho-phosphoric acid (USP-NF, BP, Ph. Eur.) pharma grade, iron(II) sulfate heptahydrate, 2,2′-Azino-bis(3-ethylbenzothiazoline-6-sulfonic acid) diammonium salt (ABTS) BioChemica and 96% ethanol were obtained from PanReac AppliChem (Darmstadt, Germany).

Peroxidase from horseradish, cytochrome c from equine heart (Cytc), Bradford reagent and bovine serum albumin (BSA) standard were obtained from Sigma-Aldrich (part of Merck KGaA, Darmstadt, Germany).

Boric acid and 2,6-Dichlorophenolindophenol (DCIP) sodium salt hydrate were obtained from Fisher Scientific, Honeywell Fluka (Schwerte, Germany). Zeocin^TM^ was obtained from Thermo Fisher Scientifc, Invitrogen (Waltham, MA, USA). Antifoam Struktol J673 (antifoam) was obtained from Schill + Seilacher (Böblingen, Germany).

### 2.2. Strains and Proteins

*K. phaffii* strains with the respective gene of interest and Zeocin^TM^ resistance were not generated in this study, but cryogenic glycerol stocks were kindly provided from different sources. *K. phaffii* strains SMD1168H Mut^+^ (protease-deficient [56]), BSYBG11 Mut^S^ (wild-type) and ATUM PPS 9011 Mut^S^ (wild-type) were used. The industrial enzymes galactose oxidase ((GalOx; (monomeric, 1× copper cofactor), horseradish peroxidase isoenzyme A2A ((HRP); monomeric, 2x calcium cofactor, iron heme group) and flavocytochrome ((FC); and monomeric, 1× FAD in dehydrogenase domain (DH), 1× heme in iron heme domain, DH domain linked to iron heme domain) were used. GalOx is often applied in biosensors for the detection of lactose in dairy products as well as for medical diagnostics [57]. HRP finds a wide variety of applications such as biosensors, coupled enzyme assays, wastewater treatment, medical diagnostics and cancer treatment [58]. The respective FC protein is of proprietary nature and is therefore not described in detail.

SMD1168H with GalOx was previously constructed with a pPICZα-C vector (Invitrogen, CA, USA) by insertion of the respective genes into the alcohol oxidase 1 (AOX1) locus [57]. BSYBG11 with codon-optimized HRP was also available in our group and expression was possible by induction with methanol [59]. The proprietary strain ATUM PPS 9011 with FC was kindly provided by an industrial partner and expression was inducible by methanol.

### 2.3. Leakage of Elements from Cultivation Vessels

Element leakage experiments were performed for three different materials. Leakage was investigated in standard borosilicate glass shake flasks, in plastic Ultra Yield™ Flasks (Thomson Instrument Company, Oceanside, CA, USA) and in a stainless steel bioreactor Biostat^®^ Cplus 10 L (Sartorius AG, Goettingen, Germany). Prior to experiments, the respective vessels were vigorously cleaned with MQ, then autoclaved with MQ. The autoclaved MQ was discarded, the vessels were again cleaned with fresh MQ and then filled with buffer. Buffer was chosen instead of growth medium to improve detectability of leaking elements in the subsequent analytics. A 0.5 M potassium phosphate buffer at pH 5.0 was used, because *K. phaffii* cultivations are usually performed between pH 5–6 under high ionic concentrations [15]. The results give an estimate on what to expect in terms of element leakage.

A defined volume of buffer (0.05 L in glass; 0.16 L in plastic; 7.20 L in steel) was added to the respective vessels and an initial sample (t_0_) was taken. Then, filled vessels were autoclaved for 20 min at 121 °C and incubated at 30 °C under shaking (230 rpm) or stirring (300 rpm) for 142 h under sterile conditions. Additionally, the bioreactor was aerated with dried air at 2 L·min^−1^. After 142 h, the respective vessels were sampled (t_142_) and the final volumes were measured (0.043 ± 0.002 L in glass; 0.135 ± 0.005 L in plastic; 6.58 ± 0.10 L in steel). This was necessary to account for lost water during the process. Samples were stored in polypropylene containers until analysis. Experiments were performed in duplicates and samples t_0_ and t_142_ were analyzed by inductively coupled plasma optical emission spectrometry (ICP-OES) as explained below.

### 2.4. Preculture and Cultivation Media

#### 2.4.1. Precultures

Precultures were carried out in shake flasks (either plastic or borosilicate glass) in YNB-medium containing 752 mL·L^−1^ MQ, 43.98 mL·L^−1^ D-Glucose monohydrate stock (500 g·L^−1^), 100 mL·L^−1^ of a 1 M potassium phosphate buffer pH 6.0 stock (26.13 g·L^−1^ K_2_HPO_4_, 115.67 g·L^−1^ KH_2_PO_4_), 100 mL·L^−1^ YNB stock (134 g·L^−1^ YNB), 4 mL·L^−1^ Biotin stock (100 mg·L^−1^ D-Biotin in 0.05 M NaOH); 50 µg·mL^−1^ of a Zeocin stock (100 g·L^−1^ Zeocin^TM^). Cells were grown from cryogenic stocks at 30 °C and shaken at 230 rpm for 24 h. The liquid volume was set to 10–20% of the working volume of the shake flask to assure proper aeration. Depending on the respective cultivation, precultures were performed either in laboratory glass shake flasks or in plastic Ultra Yield™ flasks (Thomson Instrument Company, Oceanside, CA, USA).

#### 2.4.2. Bioreactor Cultivation Media

The standard defined medium for high cell density growth and protein production in literature is defined as BSM supplemented with PTM1 solution (“Invitrogen Pichia Fermentation Process Guidelines”) [15,16]. It is known that addition of PTM1 solution leads to precipitation of phosphate salts at pH 5–6 [15]. The formation of phosphate precipitates occurs after autoclavation of BSM when PTM1 solution is added but does neither hinder cell growth nor the process itself [15]. The herein used medium was based on a partially optimized, simpler BSM that contained less salts and phosphoric acid to decrease precipitation. Previous studies from our laboratory showed that this BSM composition hindered neither biomass growth nor recombinant protein production with *K. phaffii* [60,61]. However, we increased the magnesium content in the current study to avoid limitation. Therefore, BSM for cultivations contained: 51.6 g·L^−1^ Glycerol, 10.79 mL·L^−1^ 85% ortho-phosphoric acid, 0.18 g·L^−1^ calcium sulfate dihydrate, 13.62 g·L^−1^ potassium sulfate, 4.48 g·L^−1^ magnesium sulfate heptahydrate, 4.13 g·L^−1^ potassium hydroxide in MQ. Additionally, 100 mL·L^−1^ preculture, 4.35 mL·L^−1^ PTM1 solution and base were added aseptically to the cultivation vessel after autoclavation. PTM1 solution contained: 3.84 g·L^−1^ copper(II) sulfate, 0.08 g·L^−1^ sodium iodide, 3 g·L^−1^ manganese sulfate monohydrate, 0.02 g·L^−1^ boric acid, 0.91 g·L^−1^ cobalt dichloride hexahydrate, 0.2 g·L^−1^ sodium molybdate dihydrate, 20 g·L^−1^ zinc dichloride, 65 g·L^−1^ iron(II) sulfate heptahydrate, 5 mL·L^−1^ sulfuric acid, 0.2 g·L^−1^ D-Biotin in MQ. For experiments without cobalt and boric acid, a separate PTM1 solution was prepared without the respective substances. Feed for the fed-batch phase contained either 500 g·L^−1^ or 750 g·L^−1^ glycerol in MQ. For feeding in the induction phase, pure methanol was used and 12.5% NH_4_OH solution was used for pH adjustment. For practical reasons, methanol and base had to be stored in borosilicate glass bottles rather than plastic during cultivations.

### 2.5. Bioreactor Cultivations

Laboratory setup bioreactor cultivations were performed in 2.7 L working volume glass vessels in a 4× parallel DASGIP bioreactor system (Eppendorf AG, Hamburg, Germany) equipped with two Rushton four-blade disc turbines. Starting volumes of HRP and GalOx cultivations were 1.2 L. FC cultivations were started with a batch volume of 1.5 L. The system was equipped with a calibrated EasyFerm Plus pH probe (Hamilton, Reno, NV, USA) and a fluorescence dissolved oxygen (dO_2_) electrode Visiferm DO425 (Hamilton, Reno, NV, USA). Calibration of the pH probe was performed prior to autoclavation. Calibration of the dO_2_ probe was performed after autoclavation with N_2_ and dried air at cultivation conditions after base and PTM1 were added. The cultures were aerated with dried air and pure oxygen was added, if necessary, to maintain a respective dO_2_ setpoint (vide infra). Furthermore, a dO_2_ control cascade was employed with constant stirring and variable aeration until 2 vvm. Offgas CO_2_ and O_2_ of the cultures were measured with a DASGIP^®^ GA gas analyzer (Eppendorf, Hamburg, Germany). All process parameters were adjusted and logged by the process information management system DasGip Control (Eppendorf, Hamburg, Germany).

Pilot setup bioreactor cultivations were performed in a stainless steel Biostat^®^ Cplus 10 L (Sartorius AG, Goettingen, Germany) with a starting volume of 6.06 L. Cultures were aerated with dried air. The dO_2_ was monitored with a fluorescence electrode Visiferm DO425 (Hamilton, Reno, NV, USA). Calibration of the pH probe was performed prior to autoclavation. Calibration of the dO_2_ probe was performed after autoclavation with N_2_ and dried air at cultivation conditions after base and PTM1 were added. Furthermore, a dO_2_ control cascade was employed with constant stirring and variable aeration until 2 vvm. The pH was monitored with an EasyFerm electrode (Hamilton, Reno, NV, USA) and maintained by addition of 12.5% NH_4_OH. Base consumption was determined gravimetrically. Offgas of the cultures was measured using an infrared cell for CO_2_ and a ZrO_2_ sensor for O_2_ concentration (Blue Sens Gas analytics, Herten, Germany). All process parameters were adjusted and logged by the process information management system Lucullus (Biospectra, Schlieren, Switzerland).

#### 2.5.1. Batch, Fed-Batch and Induction PHASE

Batch media were inoculated with preculture to start the cultivation. Batch cultivations were performed at 30 °C, pH were adjusted with 12.5% NH_4_OH to pH 6.0 and dO_2_ was kept above 30%. The end of the initial batch phase and therefore complete glycerol consumption was indicated by an increase in dO_2_, a drop in offgas CO_2_ and an increase in offgas O_2_. After the batch phase, an exponential feed-forward fed-batch phase yielded a dry cell weight (DCW) of approximately 60 g·L^−1^ by feeding a glycerol solution (500–750 g·L^−1^ in MQ). Fed-batch phase conditions were again 30 °C, pH 6.0 and a dO_2_ control above 30%. For the methanol induction phase, the glycerol feed was stopped and the temperature (GalOx = 25 °C; HRP = 25 °C; FC = 30 °C) and the respective dO_2_ setpoint (GalOx = 30%; HRP = 30%; FC = 1%) was changed. The respective temperature and dO_2_ setpoints were used according to [57] for GalOx, [59] for HRP and based on communication with an industrial partner for FC. Then, a 0.5% w/w methanol pulse was added to adapt cells to the new substrate. After adaptation, as indicated by a rise in dO_2_, methanol feeding was started in a pulsed or exponential feeding manner. Exponential feeding was targeted between a specific growth rate on methanol (µ_MeOH_) of 0.015–0.020 h^−1^, due to increased protein productivity in this range [9]. Pulsed feeding represented the dynamic methanol need of the cells, due to automatic methanol addition through a 0.5% (*w/v*) pulse when dO_2_ increased again, which marked methanol depletion. Therefore, cells grew under methanol excess during pulsed feeding around their maximum specific growth rate on methanol (µ_max, MeOH_). Induction phase was terminated around an optical density at 600 nm (OD_600_)~250 AU that marked a DCW between 80–90 g·L^−1^.

#### 2.5.2. Description of Single Cultivations

Ten high cell density cultivations (F1–F10) were performed in this study with different enzymes (Table 1). All cultivations were done in equally prepared media that only differed in the used PTM1 solution with or without cobalt dichloride (Co) and boric acid (BA). Although bioreactor cultivations were performed in a highly controlled environment, cultivations F2–F4 represent triplicates to investigate reproducibility. F7 and F8 are used as representative cultivations for ICP-OES and flow cytometry analyses. F9 and F10 were performed to show how depletion of Co and BA affects cultures in different bioreactor setups as no boron leakage from the vessel happens in this setup. Pulsed feeding was not possible for the stainless steel setup.

#### 2.5.3. Cultivation Sample Preparation

The OD_600_ of cultivation broths was measured with a spectrophotometer (Genesys 20; ThermoFisher Scientific, Vienna, Austria) in triplicates. DCW analysis was performed in triplicates by centrifugation of 5 mL cell suspension at 4000 g at 4 °C for 10 min. The supernatant was sterile filtered with a PVDF membrane and stored at 4 °C. The remaining cell pellet was washed with 5 mL of a 0.9% NaCl solution and centrifuged again. The washing supernatant was discarded and the pellet was dried at 105 °C for at least 72 h. Enzymatic measurements, protein concentration measurements and ICP-OES analyses were performed with the respective sterile supernatants. Flow cytometry measurements were performed directly after sampling.

### 2.6. Data Analysis

Although bioreactor cultivations were performed in a controlled environment, human interaction can be a source of uncertainty. Therefore, we performed triplicate cultivations F2–F4 to investigate overall process reproducibility by calculating the average absolute error (Θ) of each variable by Equations (1) and (2). This gave an approximate indication on potential error sources during bioreactor cultivations. Values of Θ < 10% represent good reproducibility. Furthermore, along with the observed standard deviations for triplicate measurements, the errors were propagated to the specific rates and the yield coefficients. Due to the manual curve fitting to the CO_2_ offgas signal, an error of 10% was estimated for the calculation of µ_max, Gly_.

(1) $$\mathrm{average}\;\mathrm{mean}\;\left({\bar{x}}_{\mathrm{ave}}\right)=\frac{\sum_{i=1}^{n}{\bar{x}}_{i}}{n}$$

(2) $$\Theta =\left(\frac{\sum_{i=1}^{n}\left|{\bar{x}}_{\mathrm{ave}}-{\bar{x}}_{i}\right|}{n}\right)\times \frac{100}{\bar{x_{ave}}}$$

i = respective cultivation of triplicate$;\;{\bar{x}}_{i}$ = calculated average of respective variable in cultivation i.

### 2.7. High Pressure Liquid Chromatography

Concentrations of glycerol, methanol and metabolites (ethanol, lactate, formate, succinate, acetate) were determined in cell-free samples by high-pressure liquid chromatography (HPLC) (U3000, ThermoFisher Scientific, Waltham, MA, USA) equipped with a Supelco guard column and a Supelco gel C-610H ion-exchange column (Sigma-Aldrich, Merck KGaA, Darmstadt, Germany) and a refractive index detector (Agilent Technologies, Santa Clara, CA, USA). The mobile phase was 0.1% H_3_PO_4_ with a constant flow rate of 0.5 mL·min^−1^ and the system was run isocratically. Calibration was done by measuring standards in the range of 0.1–10 g·L^−1^.

Potential FC degradation (linker degradation between DH domain and iron heme domain) was measured with a size-exclusion (SEC) column from Waters GmbH (XBridge^®^ BEH200A SEC 3.5 µm; Austria) at 280 and 404 nm. Absorption at 404 nm at different retention times made differentiation between whole FC and free heme-domain possible. The integral of the respective absorption peaks at 280 nm was used to determine the ratio of free heme-domain in relation to intact FC (HDR_FC_).

### 2.8. Flow Cytometry

Cell suspensions were diluted to an OD_600_ of 1 with phosphate buffered saline (PBS) (2.65 g·L^−1^ calcium dichloride, 0.2 g·L^−1^ potassium chloride, 0.2 g·L^−1^ potassium dihydrogen phosphate, 0.1 g·L^−1^ magnesium chloride hexahydrate, 8 g·L^−1^ sodium chloride and 0.764 g·L^−1^ disodium hydrogen phosphate dihydrate at pH 6.5). Then, 0.5 mL were incubated for 10 min in the dark with 0.5 µL of a 20 mM propidium iodide (PI) stock in dimethyl sulfoxide (Sigma-Aldrich, Merck KGaA, Darmstadt, Germany) and 5 µL of a 12 mM fluorescein diacetate (FDA) stock in acetone (Sigma-Aldrich, Merck KGaA, Darmstadt, Germany). Afterwards, 100 µL were mixed with 4.9 mL PBS for subsequent measurement with a flow cytometer (CytoBuoy, Woerden, Netherlands) with two forward scatter (FSC), one sideward scatter (SSC) and two fluorescence channels (green, red) [62]. The data were evaluated with the Cytoclus3 program (CytoBuoy, Woerden, Netherlands). Measurements were performed in triplicates. Data analysis was performed as shown elsewhere [63]. PI is not able to cross the membrane of healthy cells and therefore interacts with cells that are impaired, which were termed “viability declined” cells that showed increased red fluorescence (see also [64]). Furthermore, we looked for agglomerating yeast cells, termed “cell clusters” (see [63]).

### 2.9. Protein Purification

#### 2.9.1. GalOx

Next, 250 mL of filtrated final cultivation supernatant were ultra- and diafiltrated with 20 mM NaH_2_PO_4_, 500 mM NaCl, pH 7.5 on a mini-TFF membrane (Pall Corporation Minimate™ TFF Capsule, Omega 10K) at room temperature to a final volume of ~8 mL. No further purification was performed since cultivation samples contained a high amount of GalOx. Samples were sterile filtrated with regenerated cellulose (RC) filters and stored at 4 °C.

#### 2.9.2. HRP

In the next stage, 500 mL of the filtrated final cultivation supernatant were ultra- and diafiltrated with a mini-TFF membrane (Pall Corporation Minimate™ TFF Capsule, Omega 10K) with buffer A (50 mM Tris-HCl, pH 8.0) to obtain a final volume of ~8 mL. Afterwards a negative anion exchange chromatography (negative-AIEX) step with a DEAE-Sepharose FF column (GE Healthcare, Vienna, Austria) was performed on an Äkta pure (GE Healthcare, Vienna, Austria). The column was equilibrated with 10 CV buffer A, then the sample was loaded at 156 cm·h^−1^. For elution, 100% buffer B (50 mM Tris-HCl, 2M NaCl, pH 8) was reached through a linear gradient in 20 min at 156 cm·h^−1^. Absorbance was monitored at 214 nm, 280 nm and 404 nm. This yielded highly glycosylated HRP in the flowthrough and bound host cell impurities on the column [59]. The flow through was collected, concentrated and rebuffered with a centrifuge filter (5 kDa cut off) with storage buffer (50 mM Tris-HCl; 150 mM NaCl; pH 8). Samples were sterile filtrated with regenerated cellulose (RC) filters and stored at 4 °C.

#### 2.9.3. FC

Filtrated cultivation supernatant was ultra- and diafiltrated with a mini-TFF membrane (Pall Corporation Minimate™ TFF Capsule, Omega 10K) into AIEX buffer A (25 mM sodium citrate pH 6.0). Then, a proprietary purification step-chain was used. Briefly, an AIEX step with a DEAE-Sepharose FF column (GE Healthcare, Vienna, Austria) was performed on an Äkta pure (GE Healthcare, Vienna, Austria) and elution was done with AIEX buffer B (25 mM sodium citrate pH 6.0; 1 M NaCl). The elution fraction was used for a subsequent precipitation step with saturated (NH_4_)_2_SO_4_ and centrifugation to apply the supernatant on a Capto-Phenyl hydrophobic interaction chromatography (HIC) column (GE Healthcare, Vienna, Austria) on an Äkta pure (GE Healthcare, Vienna, Austria). Loading of the HIC column was done with HIC buffer A (25 mM sodium citrate pH 6.0; 20% saturated (NH_4_)_2_SO_4_) and elution with AIEX buffer A. Again, the elution fractions were pooled. Absorbance was monitored for both purification steps at 280 nm, 370 nm and 404 nm. The HIC Pool was rebuffered into 1 mM phosphate buffer pH 7.4 with a mini-TFF membrane (Pall Corporation MinimateTM TFF Capsule, Omega 10K) and concentrated with a centrifuge filter (5 kDa cut off). Samples were sterile filtrated with regenerated cellulose (RC) filters and stored at 4 °C.

### 2.10. Protein Concentration and Activity

#### 2.10.1. Protein Concentration

Measurements were performed in triplicates using a microplate reader (Infinite M200 pro; Tecan Austria GmbH, Groedig, Austria). The assay used Bradford reagent and was calibrated through a standard row with 0.05 g·L^−1^ to 0.4 g·L^−1^ BSA; 190 µL Bradford reagent was added to 10 µL diluted or undiluted protein solution and incubated at room temperature for 10 min in the dark. Afterwards, absorbance was measured at 595 nm.

#### 2.10.2. Enzyme Activity

Activity of GalOx in samples was measured after incubation with 0.5 mM CuSO_4_ for 24 h at 4 °C [58]. The GalOx activity assay was performed with 110 µl NaH_2_PO_4_ buffer (100 mM, pH 7.5), 20 µL ABTS reaction solution (14.7 mg ABTS in 2.4 mL NaH_2_PO_4_ buffer with 100 µL peroxidase stock solution), 60 µL D-Galactose solution (1 M in NaH_2_PO_4_ buffer) and 10 µL of the accordingly diluted sample. Peroxidase solution consisted of 1.5 mg peroxidase from horseradish in 1 mL 50 mM TRIS; pH 7.5; 1 M (NH_4_)_2_SO_4_ filled up to 2.5 mL with 50 mM TRIS buffer at pH 7.5. The measurement was performed at 420 nm and 30 °C for 90 s. Volumetric activity was calculated through Equation (3). The millimolar extinction coefficient of ABTS at 420 nm ***ε***_420_ is 36 mM^−1^·cm^−1^.

HRP activity assay was performed with 175 µL ABTS working solution (8 mM in 50 mM phosphate citrate buffer pH 5 (Na_2_HPO_4_ + 2 M citric acid to pH 5), 5 µL accordingly diluted enzyme sample (dilution buffer 20 mM BisTris/HCl pH 7) and 20 µL H_2_O_2_ (0.034% in MQ). The reaction was started by addition of H_2_O_2_ and increase in absorbance was measured at 420 nm at 30 °C for 120 s. Volumetric activity was calculated through Equation (3). The millimolar extinction coefficient of ABTS at 420 nm ***ε***_420_ is 36 mM^−1^·cm^−1^.

The activity of FC was measured through two different activity assays. Cytc activity assay was used to assay activity of the functionally, whole enzyme [65]. The assay was performed with 104 µL PBS (11 mM, pH 7.4), 60 µL Glucose solution (1M D-Glucose in PBS, pH 7.4), 16 µL Cytc solution (1 mM Cytc in MQ) and 20 µL accordingly diluted enzyme solution. The measurement was performed at 550 nm and 30 °C for 5 min. Volumetric activity was calculated through Equation (3). The millimolar extinction coefficient of Cytc in the oxidized state at 550 nm ***ε***_550_ is 19.6 mM^−1^·cm^−1^. The DCIP activity assay measured the activity of the whole enzyme as well as the DH domain activity [65]. The assay was performed with 100 µL PBS, 60 µL Glucose solution, 20 µL DCIP solution (3 mM (87.03 mg DCIP dissolved in 10 mL 96% ethanol at 50 °C and filled up to 100 mL with MQ)) and 20 µL accordingly diluted enzyme solution. The decrease in absorbance was measured at 520 nm at 30 °C for 5 min. Volumetric activity was calculated through Equation (3). The millimolar extinction coefficient of DCIP in the oxidized state at 520 nm ***ε***_520_ is 6.9 mM^−1^·cm^−1^.

The volumetric activity (Activity) was calculated with Equation (3), where V_t_ is the total assay volume (200 µL), df is the dilution factor, vs. is the respective sample volume, ε_λ_ is the respective millimolar extinction coefficient and 0.52 cm is the length of the light path.

(3) $$\mathrm{Activity}\;[U\cdot {\mathrm{mL}}^{-1}]=\Delta \mathrm{Abs}\cdot \min^{-1}*\frac{V_{t}*\mathrm{df}}{0.52\;\mathrm{cm}*V_{S}*\varepsilon_{\lambda }}$$

### 2.11. Enzyme Kinetics and Thermal Stability

All enzyme kinetics and thermal stability analyses were done in triplicates. However, the specific activity was not comparable due to differences in product purities. Thus, a normalized v_max_ (norm-v_max_) was used to allow comparability. This can be found in the Supplementary Materials. For GalOx, a specific activity of 1000 U·mg^−1^ was used, for HRP 100 U·mg^−1^ and for FC 10 U·mg^−1^.

For the GalOx enzyme kinetic measurements, D-Galactose concentrations of 300 mM, 200 mM, 100 mM, 50 mM, 30 mM, 20 mM, 10 mM and 3 mM were used. Enzyme thermal stability measurements were performed by incubation of the enzyme solution at 60 °C for 30 s, 1, 2, 3, 4, 5, 10 and 30 min and immediate cooling on ice. Then, residual enzymatic activity was measured as described above.

For the HRP enzyme kinetic measurements, the ABTS concentration was varied to reach final ABTS concentrations of 7 mM, 5 mM, 3 mM, 1 mM, 0.4 mM, 0.2 mM, 0.1 mM and 0.05 mM. Enzyme stability measurements were performed by incubation of the enzyme solution at 60 °C for 30 s, 1, 2, 3, 4, 5, 10 and 30 min and immediate cooling on ice. Then, residual enzymatic activity was measured as described above.

For the FC enzyme kinetic measurements, D-Glucose concentration was varied to reach final D-Glucose concentrations of 300 mM, 200 mM, 100 mM, 50 mM, 30 mM, 20 mM, 10 mM and 3 mM. Enzyme stability measurements were performed by incubation of the enzyme solution at 60 °C for 30 s, 1, 2, 3, 4, 5, 10 and 30 min and immediate cooling on ice. Then, residual enzymatic activity was measured as described above.

### 2.12. ICP-OES Measurements

The REACH regulation does not provide guidance on the methods for detection of elements. ICP-OES was used in this study. The following elements with their respective limit of detection (LOD) in parenthesis as mg·L^−1^ were investigated: B (0.03), Ca (0.156), Co (0.03), Cu (0.01), Fe (0.03), K (0.32), Mg (0.0005), Mn (0.0024), Mo (0.10) and Zn (0.02). ICP-OES for simultaneous, multi-element analysis was carried out on an iCAP 6500 series spectrometer (Thermo Scientific, Waltham, MA, USA) coupled to an ASX-520 auto sampler (Teledyne Cetac, Omaha, NE, USA). Instrumental parameters are described in Supplementary Table S1. Samples were automatically measured in triplicates by the device, which yielded a relative standard deviation (RSD) around 0.5%. The instrument was equipped with a standard sample introduction set, consisting of a concentric nebulizer and a cyclonic spray chamber. The conditions used for determination of background corrected emission signals are also presented in Supplementary Table S1. Two sensitive and non-interfered emission lines per element were used, one line for quantification, the second one for quality control. Samples were stored at 4 °C until measurement. The sample solutions were diluted 1:10 with 1%-HNO_3_ (*v/v*), for matrix adjustment; thereby Europium with a concentration level of 1.0 mg·L^−1^ has been added as internal standard to all samples. Ca, K, or Mg were analyzed using a sample dilution of 1:500 (m/m), since these elements were known to be present with higher concentrations in the cell-free cultivation samples. Quantification was done via external calibration with aqueous standard solutions using Europium as internal standard. High purity water was obtained by purifying de-ionized water with an Easypure 2 system (Thermo Scietific Barnstead, Waltham, MA, USA). Nitric acid was of p.a. grade purity (Merck, Darmstadt, Germany). A certified stock solution of Europium (Specpure ICP standard, Alfa Aesar, Kandel, Germany), Certipur multi-element VIII and Molybdenum single element ICP-standard (Merck KGgA, Darmstadt, Germany) were used for method development and quantification of sample signals. Working solutions were prepared by the dilution of stock solutions with 1.0% nitric acid (*v/v*) prior to use.

### 2.13. N-Glycosylation Profiling

Next, 30 μg of each protein were transferred into a 1.5 mL screw cap micro-tube. HRP samples were brought to a final concentration of 2.5 M urea and incubated for 10 min at 95 °C (to unfold the protein). FC samples required no reduction. Then, 1 µL PNGase F (1 Unit) was added per sample and incubated at 37 °C overnight. The released glycans were dried in a speed-vac and reconstituted in 90 µL 100 mM ammonium bicarbonate buffer and 10 µL sodium borohydride solution was added and incubated over night at room temperature.

The digested glycans were purified using HyperSep Hypercarb SPE 10 mg cartridges (Thermo Scientific, Austria (Art. No 60302606)). For that purpose, the cartridges were pre-treated with 500 µL SPE elution solution and twice with 500 µL ammonium formate solution. The samples were applied to the cartridges and washed twice with ammonium formate solution. The elution of bound glycans was performed with 500 µL SPE elution solution per sample. The samples were dried in a speed vac, dissolved in 20 µL MQ and subjected to LC-ESI-MS.

The digested samples/glycan mixtures were loaded on a porous graphitic carbon column (100 mm × 0.32 mm, Thermo Scientific, Vienna, Austria) using 80 mM ammonium formate buffer as the aqueous solvent. A gradient from 2 to 42% Eluent B (98 to 58% Eluent A) was developed over 20 min at a flow rate of 8 μL·min^−1^ using an Ultimate 3000 capillary flow LC. With this steep gradient, all glycans eluted within a time window of a few minutes, which facilitated depiction of the whole glycan profile. Detection was performed with iontrap MS (Bruker amazon speed ETD) (for FC analysis) and with a QTOF MS (Bruker maXis 4G ETD) (for HRP analysis), both equipped with the standard ESI source in positive ion, DDA mode (=switching to MSMS mode for eluting peaks). MS-scans were recorded (range: 150–2200 Da). Data interpretation and quantification was performed with DataAnalysis 4.0 (Bruker). Signal responses of the glycan variants detected, were in a similar range, thus comparing peak areas of the monoisotopic peaks was valid. However, for an exact/true value, standards would be needed. Relative amounts of *N*-glycans were obtained by deconvolution of the spectra and summing up the protonated, phosphorylated, ammonium adduct and sodium adduct of the different structures. The cut off value for deconvolution of the peaks was set to 1% for FC analysis and 0.6% for HRP analysis of the highest peak in the spectrum.

## 3. Results

The REACH regulation requires a reduction of harmful substances, like boric acid, cobalt dichloride and cobalt. Although standard yeast media contain concentrations that are in agreement with REACH, a complete depletion of such elements is advised. In bioprocessing, this urges the need to investigate media compositions to understand elemental requirements of cells and to mitigate potential elemental limitations.

### 3.1. Leakage of Elements, Elemental Media Composition and Elemental Consumption

#### 3.1.1. Elemental Leakage from Vessels

The concentrations of most measured elements were below the limit of detection or did not change extensively during the 142 h lasting experiment (Supplementary Table S2). Notably, the boron concentration increased on average by 0.945 mg·L^−1^ in borosilicate glassware. Furthermore, the magnesium concentration increased on average by 2.53 mg·L^−1^ in stainless steel. After taking into account the respective volumes before and after the experiments, the calculated leakage rates were 7.33 × 10^−3^ mg·L^−1^·h^−1^ for boron and 1.76 × 10^−2^ mg·L^−1^·h^−1^ for magnesium.

#### 3.1.2. Elemental Media Composition

The added concentrations for elements in BSM + PTM1 without precipitation are shown in Table 2. It is known that addition of PTM1 solution to BSM results in precipitate formation at pH between pH 5–6 [15]. Therefore, we performed ICP-OES analyses to investigate the initial concentration of elements at the start of the batch phase and to demonstrate which elements are most affected. Samples were taken directly after inoculation of cultivation F7 and F8 and resulting average values are shown in Table 2. Importantly, F8 contained PTM1 solution without Co and BA. Our results clearly show that the elements Mn, Co, Cu, and especially Fe and Zn, precipitated. The elements K and Ca were higher, which likely resulted from addition of the preculture. It was especially interesting to find that boron concentration increased extensively on average to 4.90 mg·L^−1^ compared to the added 0.02 mg·L^−1^. As borosilicate glassware leaks boron into medium over time, the additional boron resulted from incubation in glass bioreactors, from precultures that were performed in glass shake flasks and from base addition from glass bottles. This was also validated with a stainless steel bioreactor that leaks no boron and contained BSM with PTM1 depleted of boric acid and cobalt. When we measured the initial sample after inoculation with preculture from plastic shake flasks, ICP-OES analysis found only 0.06 mg·L^−1^ boron in the medium. However, this was still 3-fold higher than 0.02 mg·L^−1^ and showed that boron contamination is hard to avoid.

Importantly, the experiments in glass bioreactors indicated that addition of BA was not necessary to supplement the medium with boron, which pointed to an option to avoid the use of BA for yeast cultivations. Therefore, we further tested if leaked boron is taken up by cells.

#### 3.1.3. Elemental Consumption Analysis

**Figure 1:**
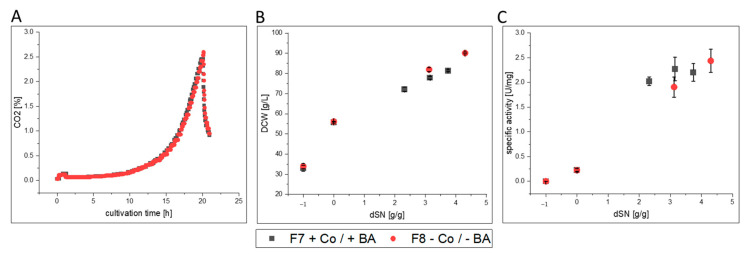
Process comparison of cultivations F7 and F8 with and without cobalt (Co) and boric acid (BA). (**A**) CO_2_ signal over time during batch phase on glycerol that mirrored growth kinetics of cells. Batch phases did not differ from each other. (**B**) Shows the DCW and (**C**) shows the specific activity of produced FC enzyme in the supernatant against the dSN (added methanol in relation to total DCW mass at induction start). In (**B**,**C**) a dSN of −1 represents the end of the batch phase and a dSN of zero represents the end of the fed-batch on glycerol.

We investigated which elements were taken up during high cell density cultivation on glycerol and methanol. Cultivation F7 was used as a representative example and ICP-OES results were compared between batch start and cultivation end (Supplementary Figure S1). Although not relevant for REACH, all investigated elements but Fe and Mo decreased until cultivation end (Supplementary Figure S1). Due to the initial precipitation of iron, its increased concentration at cultivation end likely derived from secreted free Fe ions, the secreted FC enzyme and secreted iron-cofactor host cell proteins. The small increase in Mo could result from impurities of used chemicals and highlighted that Mo might not be needed by *K. phaffii*. Importantly, the ICP-OES data showed that the initially present boron concentration of 5.30 mg·L^−1^ decreased to 3.67 mg·L^−1^ in F7. Therefore, clearly more boron than 0.02 mg·L^−1^, which would be present without boron leakage, was taken up by cells. Cobalt was also taken up and its concentration decreased from an initial 0.57 mg·L^−1^ to 0.07 mg·L^−1^. ICP-OES analysis of cultivation F8, in which no BA and Co was supplemented, showed that boron concentration decreased from an initial 4.48 mg·L^−1^ to 4.14 mg·L^−1^ and Co concentration was below the LOD at start and end of the cultivation. The found difference of boron in F7 and F8 clearly resulted from omnipresent boron leakage from glassware, which also prevented accurate calculations for boron uptake. Importantly, the initial precipitation of elements and the depletion of BA and Co hindered neither growth to high cell densities (Figure 1A,B) nor recombinant FC production (Figure 1C).

**Figure 2:**
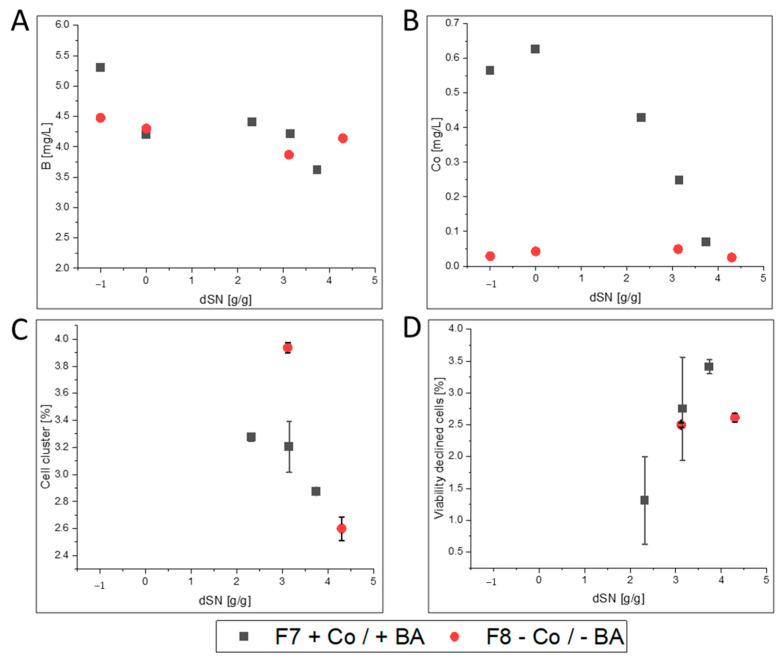
Boron and cobalt concentration with respective flow cytometry data for F7 and F8. Data are shown against the dSN (added methanol in relation to total DCW mass at induction start) with or without cobalt (Co) and boric acid (BA). A dSN of −1 represents the end of the batch phase and a dSN of 0 represents the end of the fed-batch on glycerol. ICP-OES data are shown in (**A**) for boron and (**B**) for cobalt. (**C**) Flow cytometry data for “Cell clusters” in % of total number of yeast cells in measurement. (**D**) Flow cytometry data for “Viability declined cells” in % of total number of yeast cells in measurement.

Although only a limited number of measurements were performed, the results clearly indicated that cell viability and morphology were not negatively affected during induction when Co and BA were not present in F8 compared to F7 (Figure 2). Therefore, our results indicate that cobalt was not necessary for growth and protein production and that boron leakage from borosilicate glassware was a suitable option to avoid addition of BA to the cultivation medium. Nevertheless, a deeper look into growth physiology, protein productivity and the resulting protein quality was taken.

### 3.2. Impact of Co and BA Depletion on Recombinant Protein Production in Glass Bioreactors

It is important to mention that all cultivations were performed in the controlled environment of a bioreactor, however sampling required human interaction. Therefore, cultivations F2–F4 represented triplicates to calculate the variability for each investigated variable. An average absolute error (Θ) < 10% highlighted good reproducibility (Supplementary Table S3). As expected, protein-related variables tended to higher Θ values, due to the required sample handling. Notably, the thermal stability (Τ_1/2, 60 °C_) measurement showed the highest variability with a Θ of 19.1%. This was clearly related to the used method that relied on manual sample handling rather than automation.

**Figure 3:**
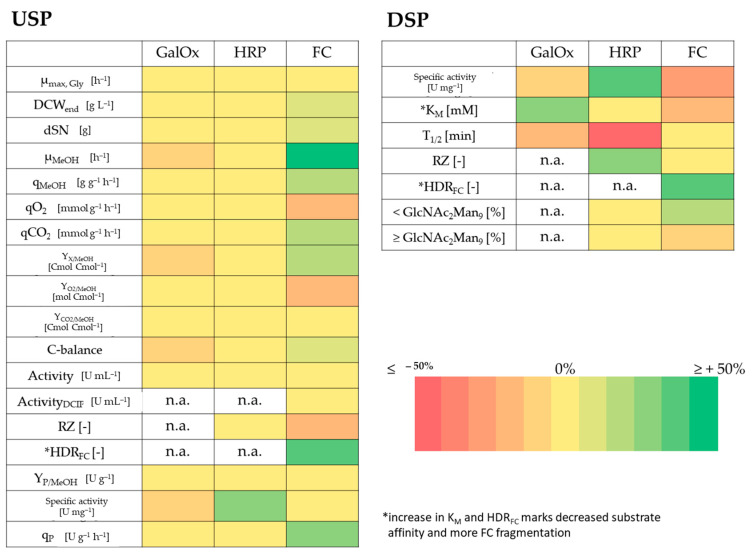
Visualization of changes of investigated variables under cobalt (Co) and boric acid (BA) depletion. The USP cultivation process data and the DSP protein quality data are compared for GalOx, HRP and FC cultivations in laboratory glass bioreactors. Data were compared with the corresponding experiments with Co and BA. The average values of F2–F4 are compared to results from F1 for GalOx. F6 is compared to F5 for HRP. F8 is compared to F7 for FC. Coloration indicates magnitude of change in %. A change between ± 10% resulted in no change in coloration. An increase in K_M_ and HDR_FC_ has to be considered as a negative impact. n.a. = not applicable; this variable was not investigated for this protein.

The 10% threshold was also used to investigate the impact of Co and BA depletion on growth physiology, protein productivity and protein quality. Although cultivation to high cell densities and protein production was clearly feasible in laboratory glass bioreactors without addition of Co and BA, a more detailed investigation was necessary. In Figure 3, the magnitude of change is shown for each investigated variable in Co and BA depleted cultivations that yielded GalOx, HRP or FC.

#### 3.2.1. GalOx

**Figure 4:**
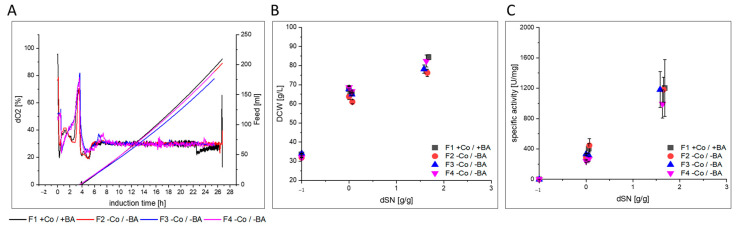
Process comparison of cultivations F1–F4 with and without cobalt (Co) and boric acid (BA). (**A**) dO_2_ signal and exponential methanol feeding over time during induction phase. Initial decrease in dO_2_ marks the addition of the methanol adaptation pulse. Second peak marks metabolization and start of methanol feeding. (**B**) Shows the DCW and (**C**) shows the specific activity of produced GalOx enzyme in the supernatant against the dSN (added methanol in relation to total DCW mass at induction start). In (**B**,**C**) a dSN of −1 represents the end of the batch phase and a dSN of zero represents the end of the fed-batch on glycerol.

Numerical values for each variable for GalOx in Figure 3 can be found in Supplementary Table S3. GalOx production was investigated under exponential methanol feeding with a Mut^+^ strain in high cell density cultivations F1–F4 (Figure 4). After an initial methanol adaptation pulse, the cells required approximately 4 h for complete consumption and adaptation to methanol (Figure 4A). In the subsequent exponential methanol fed-batch, the DCW (Figure 4B) and specific activity (Figure 4C) neither indicated a difference in growth nor in protein production. However, a detailed look on Co and BA depleted cultivations (F2–F4) showed slower growth, as indicated by a decreased µ_MeOH_ of 0.016 h^−1^ compared to 0.019 h^−1^ (Figure 3 and Supplementary Table S3). Additionally, a decreased Y_X/MeOH_ of 0.30 Cmol·Cmol^−1^ compared to 0.37 Cmol·Cmol^−1^ indicated that substrate conversion to biomass was negatively affected. We also noted that C-Balances did not close in F2–F4, which could point to undetected metabolite accumulation. However, HPLC analyses could not detect substantial metabolite accumulation in any cultivation. The additionally found decrease in specific activity in the broths of F2–F4 could indicate increased secretion of host cell proteins, but based on the found standard deviations for this variable (Supplementary Table S3), no clear conclusion was possible. Therefore, the USP was slightly negatively affected when Co and BA were not supplemented to the cultivations. However, this probably resulted from Co depletion alone, because previous ICP-OES analyses showed that boron is not limited in glass bioreactors and that leaked boron is taken up (Figure 2A). Further investigations on the respective GalOx proteins revealed that protein quality was negatively affected in F2–F4 (Supplementary Table S3 and Figure 3). However, due to the high Θ for Τ_1/2, 60 °C_, we did only consider the change in K_M_. K_M_ increased on average by 35%, which marked a clear decrease in substrate affinity of GalOx. This might be linked to differences in the intracellular protein processing machinery under depletion of Co, which is a known cofactor of yeast methionine aminopeptidases [50,51].

Therefore, excluding Co and BA from the cultivation medium did slightly affect cell physiology, but had no clear impact on productivity of the SMD1168H Mut^+^ strain producing recombinant GalOx. However, GalOx was affected in substrate affinity, which might be linked to intracellular protein processing.

#### 3.2.2. HRP

**Figure 5:**
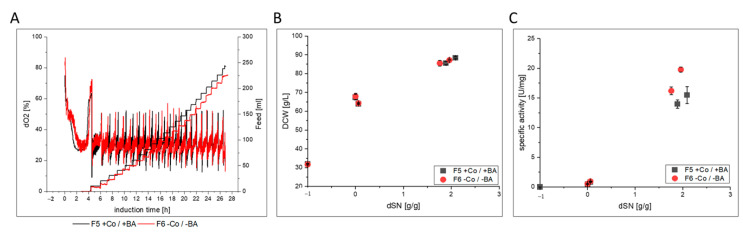
Process comparison of cultivations F5 and F6 with and without cobalt (Co) and boric acid (BA). (**A**) dO_2_ signal and pulsed methanol feeding over time during induction phase. Initial decrease in dO_2_ marks addition of methanol adaptation pulse. Second peak marks metabolization and start of methanol feeding. (**B**) Shows the DCW and (**C**) shows the specific activity of produced HRP enzyme in the supernatant against the dSN (added methanol in relation to total DCW mass at induction start). In (**B**,**C**) a dSN of −1 represents the end of the batch phase and a dSN of zero represents the end of the fed-batch on glycerol.

Numerical values for each variable from HRP in Figure 3 can be found in Supplementary Table S4. HRP production was investigated under pulsed methanol feeding with a Mut^S^ strain in high cell density cultivations F5 and F6 (Figure 5). After an initial methanol adaptation pulse, the cells required approximately 4 h for complete consumption and adaptation to methanol (Figure 5A). In the subsequent pulsed methanol fed-batch, the DCW did not indicate a clear difference in growth, when plotted against the dSN (Figure 5B). However, the specific activity was clearly higher when Co and BA where not present (Figure 5C). A detailed look on the Co and BA depleted cultivation F6 also proved that growth was not affected and cell physiology was not impaired (Figure 3 and Supplementary Table S4). The C-balances closed and no accumulation of metabolites was found. In fact, the specific activity was the only remarkable difference in the USP (Figure 3 and Supplementary Table S4). Due to the similar volumetric activity in both cultivations, less host cell proteins were secreted in cultivation F6, which resulted in greater purity of HRP in the broth. However, it was unclear why Co depletion (boron was not limited in glass bioreactors) resulted in less host cell protein secretion. Nevertheless, when we purified HRP, a number of differences were found for protein quality (Figure 3 and Supplementary Table S4). First, HRP from cultivation F6 had an increased RZ_HRP_ of 0.09 compared to 0.07 from F5. This is consistent with the higher specific activity that remained after purification. Secondly and more importantly, HRP from F6 also had a 3-fold decreased thermal stability of 3.4 min. This was highly surprising to us, because *N*-glycan profiling did not reveal a difference in *N*-glycosylation (Figure 3 and Supplementary Figure S5A,B), which is known to affect thermal stability of HRP [66]. Compared to GalOx, the K_M_ was not affected, but again it might be possible that different intracellular protein processing occurred in the Co depleted culture, which affected HRP.

Therefore, excluding Co and BA from the cultivation medium did not affect cell physiology and productivity of the BSYBG11 Mut^S^ strain producing recombinant HRP, but the HRP had a higher purity in the broth. Additionally, purified HRP from F6 was negatively affected in thermal stability, which might be linked to intracellular protein processing, because *N*-glycosylation pattern did not differ between HRP from F5 and F6.

#### 3.2.3. FC

**Figure 6:**
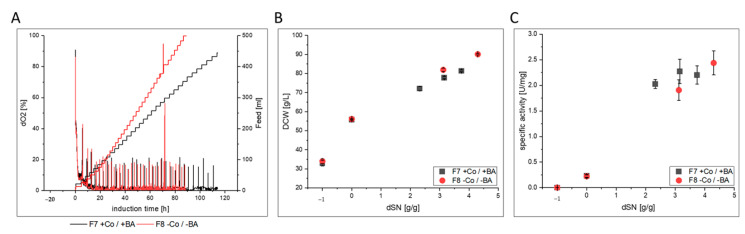
Process comparison of cultivations F7 and F8 with and without cobalt (Co) and boric acid (BA). (**A**) dO_2_ signal and pulsed methanol feeding over time during induction phase. Initial decrease in dO_2_ marks addition of methanol adaptation pulse. Second peak marks metabolization and start of methanol feeding. (**B**) Shows the DCW and (**C**) shows the specific activity of produced FC enzyme in the supernatant against the dSN (added methanol in relation to total DCW mass at induction start). In (**B**,**C**) a dSN of −1 represents the end of the batch phase and a dSN of zero represents the end of the fed-batch on glycerol.

Numerical values for each variable from FC in Figure 3 can be found in Supplementary Table S5. FC production was investigated with pulsed methanol feeding under microaerobic conditions with a Mut^S^ strain in high cell density cultivations F7 and F8 (Figure 6). After an initial methanol adaptation pulse, the cells required approximately 5.5 h for complete consumption and adaptation to methanol (Figure 5A). In the subsequent pulsed methanol fed-batch, the DCW did not indicate a clear difference in growth, when plotted against the dSN (Figure 6B) nor did the specific activity highlight a clear difference in protein production (Figure 6C). However, the feed consumption curves in Figure 6A highlighted that cells grew faster without Co and BA. It was also evident that microaerobic conditions led to an extensive decrease in growth below a µ_MeOH_ of 0.01 h^−1^ for both cultivations F7 and F8 (Supplementary Table S5). However, cultivation F8 had an approximately 60% increased µ_MeOH_ of 0.008 h^−1^, an increased q_S, MeOH_ and an increased Y_X/MeOH_, but required overall less oxygen for methanol oxidation as seen by a smaller Y_O2/MeOH_ of 0.73 mol Cmol^−1^ compared to 1.04 mol·Cmol^−1^ in F7. Cultivation F8 was also the only cultivation in which ethanol accumulation to 6.5 g·L^−1^ was found at cultivation end. During decreased activity of the oxidative phosphorylation pathway, which requires oxygen and is therefore competing with the methanol utilization pathway, cells can metabolize pyruvate to acetaldehyde and finally to ethanol, which leads to formation of crucial NAD+ from NADH. This might explain the overall lower Y_O2/MeOH_. It seemed that Co limitation induced additional stress to oxygen-limitation. Nevertheless, enzyme productivity increased by 25% in F8, although secreted FC showed slightly increased degradation and reduced cofactor loading (Figure 3). This decreased cofactor loading was not present after purification that mainly removed unloaded FC. Most importantly, purified FC from F8 had an approximately 30% higher substrate affinity and compared to F5 and F6 with HRP, the *N*-glycosylation pattern was affected. Although it is known that *N*-glycosylation decreases with decreasing µ_MeOH_ [67], cultivation F8 (higher µ_MeOH_) yielded a purified FC with a decreased glycan pattern compared to FC from F7 (Supplementary Figure S6 and Supplementary Table S5). This might point to intracellular recycling of molecules under high stress, including sugar intermediates intended for protein glycosylation.

Therefore, excluding Co and BA from the cultivation medium did affect cell physiology and productivity of the ATUM PPS 9011 Mut^S^ strain producing recombinant FC. Additionally, FC had a decreased quality in the cultivation supernatant, but after purification, a positively affected substrate affinity, which might be linked to less *N*-glycosylation, resulting in less sterical hindrance through glycans.

### 3.3. Impact of Co and BA Depletion on HRP Production in a Stainless Steel Bioreactors

**Figure 7:**
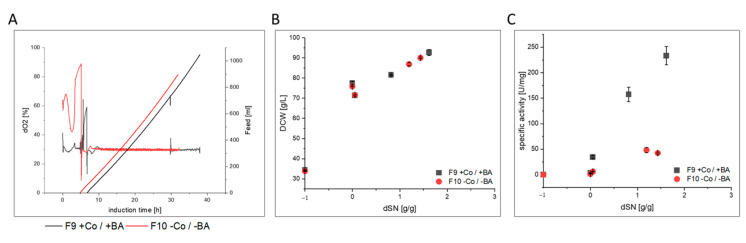
Process comparison of cultivations F9 and F10 with and without cobalt (Co) and boric acid (BA). (**A**) dO_2_ signal and exponential methanol feeding over time during induction phase. A methanol adaptation pulse was followed by methanol feeding when dO_2_ increased again. (**B**) Shows the DCW and (**C**) shows the specific activity of produced HRP enzyme in the supernatant against the dSN (added methanol in relation to total DCW mass at induction start). In (**B**,**C**) a dSN of −1 represents the end of the batch phase and a dSN of zero represents the end of the fed-batch on glycerol.

After the cultivations in glass laboratory bioreactors were analyzed, we wondered if Co and BA depletion would yield similar results in a stainless steel bioreactor. Thus, we performed experiments F9 and F10. Unfortunately, we could not perform pulsed methanol feeding due to practical reasons, and did exponential feeding instead. Surprising to us, biomass growth was not affected (Figure 7B), but HRP production was heavily impaired in cultivation F10 when Co and BA were not added (Figure 7C). In fact, the µ_MeOH_, q_MeOH_ and Y_X/MeOH_ were comparable in F9 and F10 (Supplementary Table S6). However, cells in F10 did not only secrete less active HRP, but also more host cell proteins as indicated by the 2-fold lower RZ_HRP_ and 6-fold lower specific activity at cultivation end (Supplementary Table S6). Although comparability to F5 and F6 was hardly given from a process point of view, we were astonished to find an approximately 3-fold decreased thermal stability of 4.1 min for HRP from cultivation F10, similar to F6. Additionally, the N-glycan patterns differed this time between HRP from F9 and F10 as cultivation F9 yielded HRP with decreased N-glycosylation.

Therefore, excluding Co and BA from the cultivation medium in a stainless steel vessel did not affect cell physiology, but negatively affected productivity of the BSYBG11 Mut^S^ strain producing recombinant HRP. In the light of our previous results, this was probably caused by Co depletion rather than boron limitation. Additionally, purified HRP from F10 was negatively affected in thermal stability. Although N-glycosylation was affected in F9 and F10, together with the results from F5 and F6, it seems that protein stability was rather affected by differences in intracellular protein processing than by N-glycosylation patterns.

## 4. Discussion

The REACH regulation requires the reduction of hazardous substances, like boric acid, cobalt dichloride and free cobalt. Furthermore, it is clearly mentioned that one should aim for complete avoidance of such substances, which decreases also any potential problems when handling these substances in a concentrated form in the laboratory. However, boric acid and cobalt dichloride are standard components of defined cultivation media of yeasts, because boron supports growth [43] and cobalt is found as a cofactor in yeast methionine aminopeptidases [50,51]. Therefore, complete depletion of boron and cobalt might affect the cellular machinery during recombinant protein production, which urges the need for more data.

It was already reported that borosilicate glassware leaks boron into acidic and basic environments [54]. In the current study, ICP-OES measurements verified this and we found a boron leakage of 7.33 × 10^−3^ mg·L^−1^·h^−1^ into buffer at pH 5 and 30 °C. Our further experiments with cultivation medium also underlined that different media and storage conditions lead to different boron leakage, as previously shown [54]. Importantly, this presents an interesting way to assure boron supplementation of media without the actual addition of boric acid. Nevertheless, one should consider that boron leaks as boric acid from borosilicate glass [68] and our analyses showed that leaked boron exceeded added boron by magnitudes. Therefore, one can only truly reduce the boron content of cultivation media by a strict use of non-borosilicate flasks and vessels. In fact, none of the performed experiments in this study were 100% free of boron. A recent study also showed that complete boron depletion is only hard to accomplish [43]. Importantly, this was different for cobalt, which presents a greater danger to human health. Contamination of media with cobalt might be only possible through usage of cobalt-contaminated chemicals. It should be also noted that our results indicate that molybdenum is not required for yeast growth and a recent genomic study showed that yeasts from the subphylum *Saccharomycotina*, like *S. cerevisiae* and *K. phaffii*, have lost the ability to use this metal [69]. Therefore, molybdenum can be avoided in future media recipes to improve the ecological footprint of yeast cultivations.

When we cultivated cells in media without boric acid and cobalt in glass bioreactors, high cell density cultivation was possible. However, flow cytometry analyses showed that overall cell viability decreased and cell morphology changed during methanol feeding. This was clearly not related to different media conditions, because the same trend was found with cobalt and boric acid. Importantly, it is known that methanol addition affects the viability of methylotrophic *K. phaffii* cells (e.g., [70]).

Although our data conclude that neither Mut^+^ nor Mut^S^ strains were critically affected by Co and BA depletion, a deeper investigation of growth physiology, recombinant protein productivity and the resulting protein quality yielded that cobalt depletion (boron was always present through leakage) must be investigated for each protein specifically. The most important responses from our Co and BA depleted cultivations were:

- GalOx producing cells in F2–F4 showed a slightly lower µ_MeOH_ and purified GalOx had a decreased substrate affinity.
- HRP producing cells in F6 showed a slightly decreased host cell protein secretion that led to increased specific activity in the broth. After purification, the enzyme kinetics and N-glycosylation patterns were not affected, but the thermal stability was 3-fold reduced. However, when cells were cultivated under exponential rather than pulsed methanol feeding in a stainless steel bioreactor, HRP-producing cells in F10 had an extensively decreased productivity. N-glycosylation was affected and surprisingly, HRP from F10 showed a 3-fold decrease in thermal stability, similar to HRP from F6.
- FC-producing cells in F8 showed ethanol production, faster growth and an increased productivity. The purified enzyme had increased substrate affinity and decreased glycosylation.

Although the different cultivations reacted differently to cobalt depletion, they had one aspect in common that is a change in protein quality. Due to no available -omics data on yeasts or other industrially relevant organisms under cobalt depletion, we can only speculate why protein quality was affected. Given that until now, only methionine aminopeptidases are known to use cobalt in yeasts [50,51], we assume that activity of these enzymes was reduced in cobalt-depleted cultures. It is reasonable to argue that decreased N-terminal processing can affect the overall protein fold, activity and stability of proteins. One study reported that unprocessed proteins with intact N-terminal methionine can have an extensively decreased half-life time in vivo in yeast [71]. Therefore, cobalt depletion might have a global impact on the activity and functionality of various intracellular enzymes that are also part of the posttranslational protein processing machinery.

## 5. Conclusions

Current yeast cultivation media contain substances of high concern and based on the REACH regulation, one should aim to reduce or avoid using the trace chemicals boric acid and cobalt dichloride. Neither is required for high cell density cultivation, especially because boron limitation is hardly likely to be accomplished due to omnipresent boron leakage from glassware. Furthermore, we highlight that future yeast media can also avoid molybdenum. Although not mentioned by the REACH regulation, it presents a critical heavy metal and is not required for *K. phaffii* cultivations. Finally, recombinant protein production is possible without the addition of cobalt and boric acid, but a comparison with three different enzymes showed that protein-specific investigations are necessary, because protein quality is clearly affected in bioreactor cultivations.

## Figures and Tables

**Table 1:** Overview on the performed cultivations. Cultivations were performed either in glass bioreactors (glass) or in stainless steel bioreactors (steel) with exponential (exp.) or pulsed methanol feeding. Addition of Co and BA is indicated respectively.

Cultivation	Bioreactor Type/Feeding	Strain	Target Protein	Co and BA Added	Comments
F1	glass/exp.	SMD1168H Mut^+^	GalOx	yes	
F2	glass/exp.			no	triplicates
F3	glass/exp.			no	
F4	glass/exp.			no	
F5	glass/pulsed	BSYBG11 Mut^S^	HRP	yes	
F6	glass/pulsed			no	
F7	glass/pulsed	ATUM PPS 9011 Mut^S^	FC	yes	ICP-OES; flow cytometry
F8	glass/pulsed			no	
F9	steel/exp.	BSYBG11 Mut^S^	HRP	yes	
F10	steel/exp.			no	

**Table 2:** Comparison of added and measured elemental concentrations in cultivation medium BSM+PTM1 with Co and BA. Measured values were generated through ICP-OES from cultivations F7 and F8 directly after inoculation. Average was not calculated for the element Co, because cultivation F8 contained no Co, due to the adapted PTM1 solution < L = below limit of detection.

	B	Fe	Mn	Co	Cu	Zn	Mo	Mg	Ca	K
[mg·L^−1^]										
**Added**	0.02	56.6	4.2	1.0	6.6	41.6	0.3	441.8	41.9	5933.3
**Measured**	4.90 ± 0.57	<L	2.3 ± 0.2	0.6	1.8 ± 0.3	5.5 ± 0.8	0.3 ± 0.1	468.1 ± 25.8	48.2 ± 6.8	8333.2 ± 535.3

## Supplementary Materials

The following are available online at https://www.mdpi.com/2306-5354/7/4/161/s1, Table S1. ICP-OES Instrumental parameters and analytical wavelengths in nm. Table S2. ICP-OES results from elemental leakage experiments in mg·L^−1^. Experiments were performed in duplicates in borosilicate glass (Glass) or plastic (Plastic) shake flasks or in a stainless steel bioreactor (Steel). <L = below limit of detection. Table S3. Numerical data of variables from the USP and DSP of F1–F4. Cell physiology, GalOx productivity and protein quality related variables are shown. Cultivations “Glass” were performed in a laboratory glass bioreactor. Addition of boric acid (BA) or cobalt salts (Co) is indicated with (+) or (−). For batch phase, the µ_max, Gly_ is given, all other variables are given for induction phase after adaptation to methanol. C-balance of 1 indicates completeness of data. The average of each variable from cultivations F2–F4 was also used to calculate the average absolute error Θ that was used as an indicator for variability. The value norm-vmax was calculating by normalization of vmax to a specific activity of 1000 U·mg^−1^. Table S4. Numerical data of variables from the USP and DSP of F5 and F6. Cell physiology, HRP productivity and protein quality related variables are shown. Cultivations “Glass” were performed in a laboratory glass bioreactor. Addition of boric acid (BA) or cobalt salts (Co) is indicated with (+) or (−). For batch phase, the µ_max, Gly_ is given, all other variables are given for induction phase after adaptation to methanol. C-balance of 1 indicates completeness of data. The value norm-v_max_ was calculated by normalization of v_max_ to a specific activity of 100 U·mg^−1^. Table S5. Numerical data of variables from the USP and DSP of F7 and F8. Cell physiology, FC productivity and protein quality related variables are shown. Cultivations “Glass” were performed in a laboratory glass bioreactor. Addition of boric acid (BA) or cobalt salts (Co) is indicated with (+) or (−). For batch phase, the µ_max, Gly_ is given, all other variables are given for induction phase after adaptation to methanol. C-balance of 1 indicates completeness of data. The value norm-v_max_ was calculated by normalization of v_max_ to a specific activity of 10 U·mg^−1^. Table S6. Numerical data of variables from the USP and DSP of F9 and F10. Cell physiology, HRP productivity and protein quality related variables are shown. Cultivations “Stainless steel” were performed in a pilot stainless steel bioreactor. Addition of boric acid (BA) or cobalt salts (Co) is indicated with (+) or (−). For batch phase, the µ_max, Gly_ is given, all other variables are given for induction phase after adaptation to methanol. C−balance of 1 indicates completeness of data. The value norm-v_max_ was calculating by normalization of v_max_ to a specific activity of 100 U·mg^−1^. Figure S1. Results of ICP-OES measurements from cultivation F7. Concentrations are shown at batch start (Start) and cultivation end (End) around 85 g·L^−1^ DCW. Cultivation was performed with a batch and fed-batch on glycerol to around 60 g·L^−1^ DCW and a subsequent feeding with methanol to induce recombinant FC production. Figure S2. Process diagram of dO_2_ from cultivations F1–F4 for GalOx production with and without cobalt (Co) and boric acid (BA). Batch phase is shown until ~24 h, then fed-batch on glycerol, peak indicates that feed was stopped. Then, methanol was pulsed and second peak ~43 h indicates metabolization of pulsed methanol and start of exponential methanol feeding. Figure S3. Process diagram of dO_2_ from cultivations F5 and F6 for HRP production with and without cobalt (Co) and boric acid (BA). Batch phase is shown until ~20 h, and then fed-batch on glycerol, peak ~40 h indicates that feed was stopped. Then, methanol was pulsed and second peak ~43 h indicates metabolization of pulsed methanol and start of pulsed methanol feeding. Figure S4. Process diagram of dO_2_ from cultivations F7 and F8 for FC production with and without cobalt (Co) and boric acid (BA). Batch phase is shown until ~20 h, and then fed-batch on glycerol, peak ~30 h indicates that feed was stopped. Then, pulsed methanol feeding. Figure S5. Results of LC-ESI-MS of PNGase F released N-glycans of purified HRP. The major glycoforms are high mannose type (Man8, Man9, Man9+Hex). Peaks with (*) indicate ammonium adducts. (A) HRP from F6 (B) HRP from F5 (C) HRP from F9 (D) HRP from F10. Glycoforms up to 24 residues were found (not shown). Figure S6. Results of LC-ESI-MS of PNGase F released N-glycans of purified FC. The major glycoforms are high mannose type (Man8, Man9, Man9+Hex). Peaks with lacking HexNAc were detected (H9N1–H14N1). Peaks with (*) indicate ammonium adducts. (A) Purified FC of cobalt and boron salt supplemented cultivation C1. (B) Purified FC of cobalt and boron salt depleted cultivation C2. Figure S7. Process diagram of dO_2_ from cultivations F9 and F10 for HRP production with and without cobalt (Co) and boric acid (BA). Batch phase is shown until ~24 h, then fed-batch on glycerol, peak around 43 h indicates that feed was stopped. Then, methanol was pulsed and dO_2_ increase ~50 h indicates metabolization of pulsed methanol and start of exponential methanol feeding. The dO_2_ control was set too sensitive in cultivation F9, which resulted in low visualization potential of the response to the methanol pulse.

## Abbreviations of Variables

**Θ** = average absolute error [%]; **DCW_end_** = dry cell weight at phase end [g·L^−1^]; **dSN** = added methanol in relation to total dry cell weight biomass content at induction start [g·g^−1^]; **µ_max, Gly_** = maximum specific growth rate on glycerol during batch [h^−1^]; **µ_max, MeOH_** maximum specific growth rate on methanol [h^−1^]; **µ_MeOH_** = specific growth rate on methanol [h^−1^]; **q_MEOH_** = specific methanol uptake rate [g·g^−1^·h^−1^]; **q_P_** = specific recombinant product formation rate [U·g^−1^·h^−1^]; **q_O2_** = specific oxygen uptake rate [mmol·g^−1^·h^−1^]; **q_CO2_** = specific carbon dioxide evolution rate [mmol·g^−1^·h^−1^]; **Y_X/MeOH_** = biomass per methanol yield [g·g^−1^]; **Y_O2/MeOH_** = oxygen per methanol need [mol·Cmol^−1^]; **Y_CO2/MeOH_** = carbon dioxide per methanol yield [Cmol·Cmol^−1^]; **Y_P/MeOH_** = recombinant product per methanol yield [U·g^−1^]; **C-balance** = ratio of added to found carbon in Cmol; Activity = volumetric activity of respective protein [U·mL^−1^]; **Activity_DCIP_** = volumetric activity of DH domain [U·mL^−1^]; specific activity = activity of respective protein in relation to protein content [U·mg^−1^]; **RZ_HRP_** = Reinheitszahl or purity number of HRP by ratio of 404 nm (heme) vs. 280 nm (total protein) absorbance [-]; **RZ_FC_** = Reinheitszahl or purity number of FC by ratio of 420 nm (heme and FAD) vs. 280 nm (total protein) absorbance []; **HDR_FC_** = absorption ratio at 280 nm of free heme domain in relation to intact FC by SEC HPLC [-]; **K_M_** = Michaelis-Menten constant [mM]; **v_max_** = maximum product formation rate [U·mg^−1^]; **Τ_1/2, 60°C_** = thermal half-life at 60 °C [min]; **norm-v_max_** = v_max_ normalized to a given specific activity; **vvm** = volume gas per reactor volume per min [L·L^−1^·min^−1^].
