# Supplementary material for: Depletion of Boric Acid and Cobalt from Cultivation Media: Impact on Recombinant Protein Production with Komagataella phaffii
Source: Bioengineering (Basel). 2020 Dec 13;7(4):161. doi: 10.3390/bioengineering7040161 (PMC7763993; doi:10.3390/bioengineering7040161)
Supplement: Supplementary file 1 [file bioengineering-07-00161-s001.pdf]

bioengineering

MDPI

Supplementary Materials

Depletion of Boric Acid and Cobalt from Cultivation Media: Impact on Recombinant Protein Production with *Komagataella phaffii*

Alexander Pekarsky [1], Sophia Mihalyi [1], Maximilian Weiss [2], Andreas Limbeck [2] and Oliver Spadiut [1,*]

[1] Institute of Chemical, Environmental and Bioscience Engineering, TU Wien, Gumpendorferstrasse 1a, 1060 Vienna, Austria; alexander.pekarsky@tuwien.ac.at (A.P.); sophia.mihalyi@students.boku.ac.at (S.M.)
[2] Institute of Chemical Technologies and Analytics, TU Wien, Getreidemarkt 9/164-I[2]AC, 1060 Vienna, Austria; maximilian.weiss@tuwien.ac.at (M.W.); andreas.limbeck@tuwien.ac.at (A.L.)
* Correspondence: oliver.spadiut@tuwien.ac.at; Tel.: +43-1-58801-166473

Received: 9 October 2020; Accepted: 11 December 2020; Published: 13 December 2020

Table S1. ICP-OES Instrumental parameters and analytical wavelengths in nm.

Parameter	Value	Parameter	Value
RF power	1200 W	Nebulizer flow	0.80 L min^{-1}
Sample flow	0.80 ml min^{-1}	Auxiliary flow	0.8 L min^{-1}
Observation height	12 mm	Coolant flow	15 L min^{-1}
Integration time	10 s for 1:500 samples 25 s for 1:10 samples		
Number of replicates	3		
Background correction	constant shift from analytical line		
Element	Wavelength I (nm)		Wavelength II (nm)
B	249.773		208.893
Ca	422.673		393.366
Co	238.892		228.616
Cu	324.754		324.754
Fe	259.940		239.562
K	766.490		769.896
Mg	285.213		202.582
Mn	257.610		260.569
Mo	202.030		204.598
Zn	213.856		202.548
Eu	381.967		411.970

Table S2. ICP-OES results from elemental leakage experiments in mg L^{-1}. Experiments were performed in duplicates in borosilicate glass (Glass) or plastic (Plastic) shake flasks or in a stainless steel bioreactor (Steel). < L = below limit of detection.

	B	Fe	Mn	Co	Cu	Zn	Mo	Mg	Ca
values are given at t_0 / t_{142} in mg L^{-1}									

Glass 1	0.30 / 1.36	0.05 / 0.18	0.00 / 0.00	< L / < L	<L / < L	< L / < L	< L / < L	0.01 / 0.02	< L / 0.16
Glass 2	0.29 / 1.12	0.07 / 0.08	0.00 / 0.00	< L / < L	< L / < L	< L / < L	< L / < L	0.04 / 0.16	< L / < L
Plastic 1	0.30 / 0.30	0.11 / 0.08	0.00 / 0.00	< L / < L	0.03 / 0.06	0.04 / 0.08	< L / < L	0.01 / 0.02	0.16 / 0.30
Plastic 2	0.29 / 0.27	0.06 / 0.10	0.00 / 0.00	< L / < L	0.03 / 0.07	0.02 / < L	< L / < L	0.04 / 0.03	0.20 / 0.36
Steel 1	0.24 / 0.21	0.06 / 0.14	0.00 / 0.07	< L / < L	< L / < L	0.03 / < L	< L / < L	0.34 / 2.89	0.62 / 0.32
Steel 2	0.23 / 0.20	0.05 / 0.12	0.00 / 0.07	< L / < L	< L / < L	0.03 / < L	< L / < L	0.33 / 2.84	0.71 / 0.34

Table S3. Numerical data of variables from the USP and DSP of F1 – F4. Cell physiology, GalOx productivity and protein quality related variables are shown. Cultivations "Glass" were performed in a laboratory glass bioreactor. Addition of boric acid (BA) or cobalt salts (Co) is indicated with (+) or (−). For batch phase, the $\mu_{max, Gly}$ is given, all other variables are given for induction phase after adaptation to methanol. C-balance of 1 indicates completeness of data. The average of each variable from cultivations F2 – F4 was also used to calculate the average absolute error Θ that was used as an indicator for variability. The value norm-v_{max} was calculating by normalization of v_{max} to a specific activity of 1,000 U mg^{-1}.

	**Glass +Co / +BA (F1)**	**Glass -Co / -BA (F2 – F4)**	**Average of F2 – F4**	**Θ of F2 – F4 [%]**
**USP**				
$\mu_{max, Gly}$ [h^{-1}]	0.281 ± 0.028	0.279 ± 0.028<br>0.282 ± 0.028<br>0.282 ± 0.028	0.281	0.5
DCW_{end} [g L^{-1}]	84.4 ± 1.3	78.5 ± 0.4<br>78.2 ± 2.2<br>82.4 ± 3.1	79.6	2.3
dSN [g g^{-1}]	1.68 ± 0.10	1.89 ± 0.09<br>1.58 ± 0.05<br>1.63 ± 0.03	1.70	7.5
μ_{MeOH} [h^{-1}]	0.019 ± 0.001	0.017 ± 0.001<br>0.014 ± 0.001<br>0.016 ± 0.001	0.016	7.1
q_{MeOH} [g g^{-1}h^{-1}]	0.063 ± 0.006	0.067 ± 0.009<br>0.062 ± 0.003<br>0.062 ± 0.003	0.064	3.5
q_{O2} [mmol g^{-1}h^{-1}]	1.95 ± 0.08	2.12 ± 0.08<br>1.74 ± 0.07<br>2.03 ± 0.11	1.96	7.6
q_{CO2} [mmol g^{-1}h^{-1}]	1.11 ± 0.05	1.00 ± 0.04<br>0.99 ± 0.04<br>1.12 ± 0.06	1.04	5.4
$Y_{X/MeOH}$ [Cmol Cmol^{-1}]	0.37 ± 0.02	0.32 ± 0.02<br>0.27 ± 0.01<br>0.30 ± 0.02	0.30	6.0
$Y_{O2/MeOH}$ [mol Cmol^{-1}]	1.00 ± 0.01	1.01 ± 0.01<br>0.90 ± 0.00<br>1.05 ± 0.00	0.99	5.9
$Y_{CO2/MeOH}$ [Cmol Cmol^{-1}]	0.57 ± 0.00	0.48 ± 0.00<br>0.51 ± 0.00<br>0.58 ± 0.00	0.52	7.2
C−balance	0.94 ± 0.02	0.79 ± 0.03<br>0.78 ± 0.01<br>0.89 ± 0.02	0.82	5.7
Activity [U ml^{-1}]	734 ± 97	665 ± 58<br>732 ± 9<br>697 ± 30	698	3.2
$Y_{P/MeOH}$ [U g^{-1}]	6,996 ± 391	5,401 ± 732	6,609	12.2

		7,310 ± 712<br>7,116 ± 820		
specific activity [U mg^{-1}]	1,202 ± 373	979 ± 285<br>1,182 ± 237<br>1154 ± 256	1,078	6.5
q_P [U g^{-1}h^{-1}]	437 ± 39	358 ± 68<br>439 ± 73<br>452 ± 62	416	9.3
**DSP**				
specific activity [U mg^{-1}]	1,122 ± 102	1,142 ± 108<br>859 ± 50<br>958 ± 194	1,001	9.5
K_M [mM]	64.6 ± 2.1	91.1 ± 16.3<br>77.7 ± 12.9<br>93.7 ± 5.9	87.5	7.5
v_{max} [U mg^{-1}]	1,383 ± 61	1,457 ± 172<br>1,121 ± 24<br>1,185 ± 76	1,286	8.9
norm-v_{max} [U mg^{-1}]	1,151 ± 51	1,276 ± 151<br>1,300 ± 28<br>1,232 ± 79	1,270	8.9
$T_{1/2, 60°C}$ [min]	1.34 ± 0.15	0.79 ± 0.08<br>1.06 ± 0.02<br>139 ± 0.26	1.08	19.1

Table S4. Numerical data of variables from the USP and DSP of F5 and F6. Cell physiology, HRP productivity and protein quality related variables are shown. Cultivations "Glass" were performed in a laboratory glass bioreactor. Addition of boric acid (BA) or cobalt salts (Co) is indicated with (+) or (−). For batch phase, the $\mu_{max, Gly}$ is given, all other variables are given for induction phase after adaptation to methanol. C-balance of 1 indicates completeness of data. The value norm-v_{max} was calculated by normalization of v_{max} to a specific activity of 100 U mg^{-1}.

	Glass +Co / +BA (F5)	Glass -Co / -BA (F6)
**USP**		
$\mu_{max, Gly}$ [h^{-1}]	0.317 ± 0.032	0.310 ± 0.031
DCW_{end} [g L^{-1}]	88.4 ± 0.6	87.2 ± 0.3
dSN [g g^{-1}]	2.09 ± 0.09	1.96 ± 0.09
μ_{MeOH} [h^{-1}]	0.023 ± 0.001	0.021 ± 0.001
q_{MeOH} [g g^{-1}h^{-1}]	0.068 ± 0.004	0.066 ± 0.005
q_{O2} [mmol g^{-1}h^{-1}]	2.29 ± 0.03	2.24 ± 0.04
q_{CO2} [mmol g^{-1}h^{-1}]	1.26 ± 0.03	1.19 ± 0.04
$Y_{X/MeOH}$ [Cmol Cmol^{-1}]	0.42 ± 0.01	0.40 ± 0.02
$Y_{O2/MeOH}$ [mol Cmol^{-1}]	1.09 ± 0.01	1.10 ± 0.01
$Y_{CO2/MeOH}$ [Cmol Cmol^{-1}]	0.60 ± 0.01	0.58 ± 0.01
C-balance	1.02 ± 0.02	0.98 ± 0.02
Activity [U ml^{-1}]	3.3 ± 0.3	3.3 ± 0.0
RZ_{HRP} [−]	0.03 ± 0.00	0.03 ± 0.00
$Y_{P/MeOH}$ [U g^{-1}]	30.6 ± 3.6	31.4 ± 1.5
specific activity [U mg^{-1}]	15.5 ± 1.4	19.8 ± 0.4
q_P [U g^{-1}h^{-1}]	2.0 ± 0.3	2.0 ± 0.2
**DSP**		
specific activity [U mg^{-1}]	28 ± 5	39 ± 4
RZ_{HRP} [−]	0.07 ± 0.00	0.09 ± 0.01
K_M [mM]	2.11 ± 0.35	2.05 ± 0.25
v_{max} [U mg^{-1}]	36 ± 5	49 ± 2
norm-v_{max} [U mg^{-1}]	129 ± 18	126 ± 5
$T_{1/2, 60°C}$ [min]	9.3 ± 1.5	3.4 ± 0.2
< $GlcNAc_2Man_9$ [%]	17.8	18.1
≥ $GlcNAc_2Man_9$ [%]	82.2	81.9

Table S5. Numerical data of variables from the USP and DSP of F7 and F8. Cell physiology, FC productivity and protein quality related variables are shown. Cultivations "Glass" were performed in a laboratory glass bioreactor. Addition of boric acid (BA) or cobalt salts (Co) is indicated with (+) or (−). For batch phase, the $\mu_{max, Gly}$ is given, all other variables are given for induction phase after adaptation to methanol. C-balance of 1 indicates completeness of data. The value norm-v_{max} was calculated by normalization of v_{max} to a specific activity of 10 U mg^{-1}.

	Glass +Co / +BA (F7)	Glass -Co / -BA (F8)
**USP**		
$\mu_{max, Gly}$ [h^{-1}]	0.275 ± 0.028	0.272 ± 0.030
DCW_{end} [g L^{-1}]	81.4 ± 0.5	90.1 ± 0.4
dSN [g g^{-1}]	3.74 ± 0.06	4.30 ± 0.03
μ_{MeOH} [h^{-1}]	0.005 ± 0.000	0.008 ± 0.000
q_{MeOH} [g g^{-1}h^{-1}]	0.025 ± 0.003	0.032 ± 0.002
q_{O2} [mmol g^{-1}h^{-1}]	0.88 ± 0.02	0.71 ± 0.01

q_{CO2} [mmol g^{-1}h^{-1}]	0.53 ± 0.02	0.67 ± 0.02
$Y_{X/MeOH}$ [Cmol Cmol^{-1}]	0.24 ± 0.01	0.30 ± 0.01
$Y_{O2/MeOH}$ [mol Cmol^{-1}]	1.04 ± 0.01	0.73 ± 0.01
$Y_{CO2/MeOH}$ [Cmol Cmol^{-1}]	0.67 ± 0.01	0.69 ± 0.00
C−balance	0.91 ± 0.02	1.04 ± 0.01
Activity [U ml^{-1}]	3.7 ± 0.1	3.6 ± 0.1
$Activity_{DCIP}$ [U ml^{-1}]	7.1 ± 0.2	6.8 ± 0.2
RZ_{FC} [−]	0.16 ± 0.01	0.12 ± 0.01
HDR_{FC} [−]	0.05 ± 0.00	0.07 ± 0.00
$YP_{/MeOH}$ [U g^{-1}]	19.5 ± 1.7	18.7 ± 1.4
specific activity [U mg^{-1}]	2.2 ± 0.2	2.4 ± 0.2
q_P [U g^{-1}h^{-1}]	0.47 ± 0.06	0.62 ± 0.05
**DSP**		
specific activity [U mg^{-1}]	4.9 ± 0.2	3.1 ± 0.1
RZ_{FC} [−]	0.56 ± 0.02	0.59 ± 0.02
HDR_{FC} [−]	0.05 ± 0.00	0.07 ± 0.00
K_M [mM]	94.2 ± 3.7	73.2 ± 4.3
v_{max} [U mg^{-1}]	8.7 ± 0.2	5.4 ± 0.0
norm-v_{max} [U mg^{-1}]	17.8 ± 0.4	17.4 ± 0.0
$T_{1/2, 60°C}$ [min]	0.87 ± 0.00	0.80 ± 0.00
< $GlcNAc_2Man_{10}$ [%]	42.8	51.6
≥ $GlcNAc_2Man_9$ [%]	57.2	48.4

Table S6. Numerical data of variables from the USP and DSP of F9 and F10. Cell physiology, HRP productivity and protein quality related variables are shown. Cultivations "Stainless steel" were performed in a pilot stainless steel bioreactor. Addition of boric acid (BA) or cobalt salts (Co) is indicated with (+) or (−). For batch phase, the $\mu_{max, Gly}$ is given, all other variables are given for induction phase after adaptation to methanol. C−balance of 1 indicates completeness of data. The value norm-v_{max} was calculating by normalization of v_{max} to a specific activity of 100 U mg^{-1}.

	**Stainless steel +BA / +Co (F9)**	**Stainless steel -BA / -Co (F10)**
**USP**		
$\mu_{max, Gly}$ [h^{-1}]	0.297 ± 0.030	0.298 ± 0.030
DCW_{end} [g L^{-1}]	92.7 ± 1.5	90.1 ± 0.3
dSN [g g^{-1}]	1.62 ± 0.04	1.43 ± 0.00
μ_{MeOH} [h^{-1}]	0.014 ± 0.000	0.014 ± 0.000
q_{MeOH} [g g^{-1}h^{-1}]	0.044 ± 0.003	0.045 ± 0.002
qO2 [mmol g^{-1}h^{-1}]	1.68 ± 0.05	1.47 ± 0.02
qCO2 [mmol g^{-1}h^{-1}]	0.87 ± 0.02	0.74 ± 0.01
$Y_{X/MeOH}$ [Cmol Cmol^{-1}]	0.38 ± 0.01	0.38 ± 0.01
$Y_{O2/MeOH}$ [mol Cmol^{-1}]	1.23 ± 0.01	1.05 ± 0.01
$Y_{CO2/MeOH}$ [Cmol Cmol^{-1}]	0.64 ± 0.00	0.53 ± 0.00
C-balance	1.02 ± 0.02	0.91 ± 0.02
Activity [U ml^{-1}]	16.2 ± 1.2	9.0 ± 0.3
RZ_{HRP} [−]	0.08 ± 0.00	0.04 ± 0.00
$Y_{P/MeOH}$ [U g^{-1}]	145.0 ± 25.8	91.9 ± 13.5

specific activity [U mg^{-1}]	233.4 ± 17.9	42.5 ± 1.6
q_P [U g^{-1}h^{-1}]	6.3 ± 1.3	4.1 ± 0.7
**DSP**		
specific activity [U mg^{-1}]	914 ± 66	64 ± 14
RZ_{HRP} [−]	0.17 ± 0.02	0.12 ± 0.01
K_M [mM]	1.37 ± 0.12	1.88 ± 0.51
v_{max} [U mg^{-1}]	1,048 ± 60	80 ± 13
norm-v_{max} [U mg^{-1}]	115 ± 7	125 ± 20
$T_{1/2,\ 60°C}$ [min]	10.6 ± 2.1	4.1 ± 0.1
< $GlcNAc_2Man_9$ [%]	34.8	22.3
≥ $GlcNAc_2Man_9$ [%]	65.2	77.7

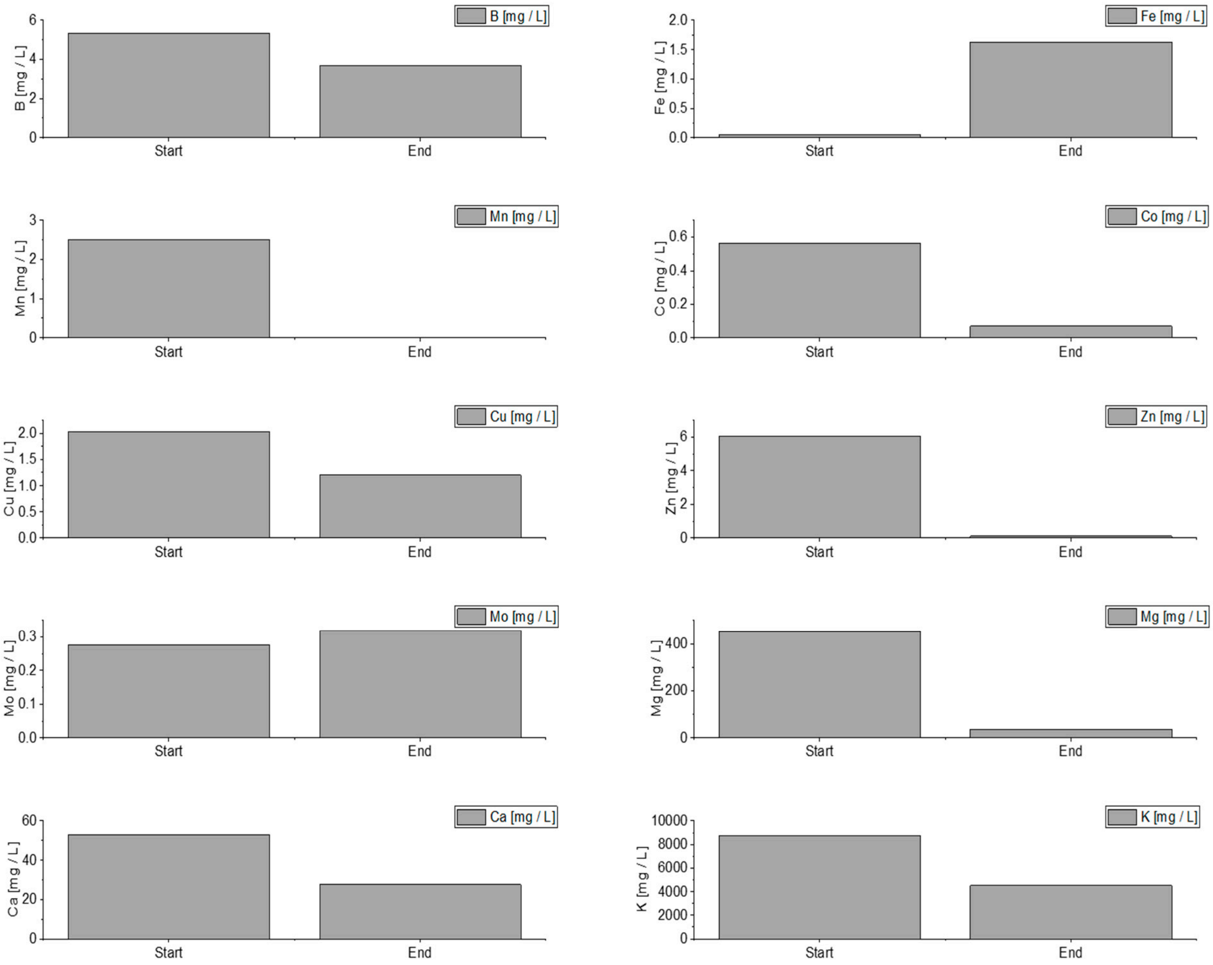

Figure S1. Results of ICP-OES measurements from cultivation F7. Concentrations are shown at batch start (Start) and cultivation end (End) around 85 g L^{-1} DCW. Cultivation was performed with a batch and fed-batch on glycerol to around 60 g L^{-1} DCW and a subsequent feeding with methanol to induce recombinant FC production.

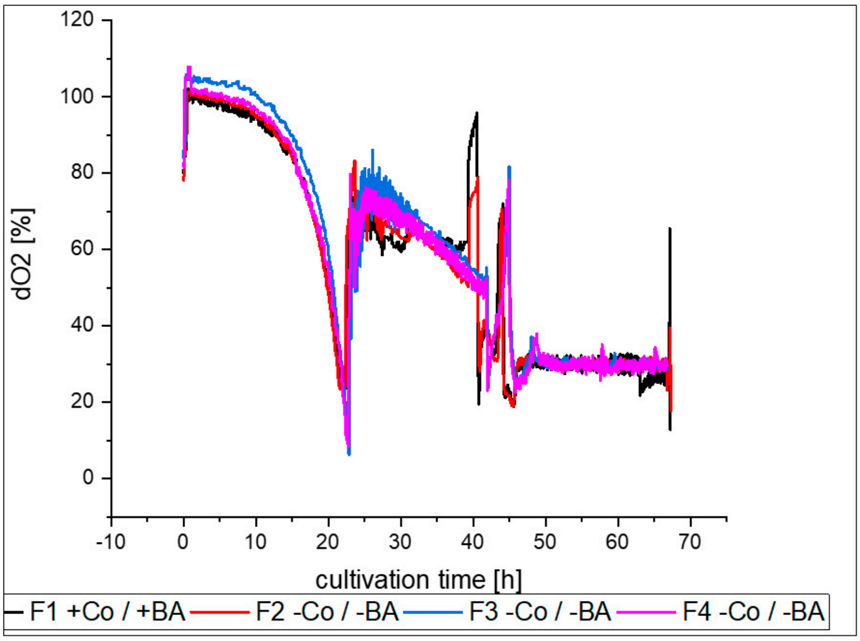

Figure S2. Process diagram of dO_2 from cultivations F1–F4 for GalOx production with and without cobalt (Co) and boric acid (BA). Batch phase is shown until ~24 h, then fed-batch on glycerol, peak indicates that feed was stopped. Then, methanol was pulsed and second peak ~43 h indicates metabolization of pulsed methanol and start of exponential methanol feeding.

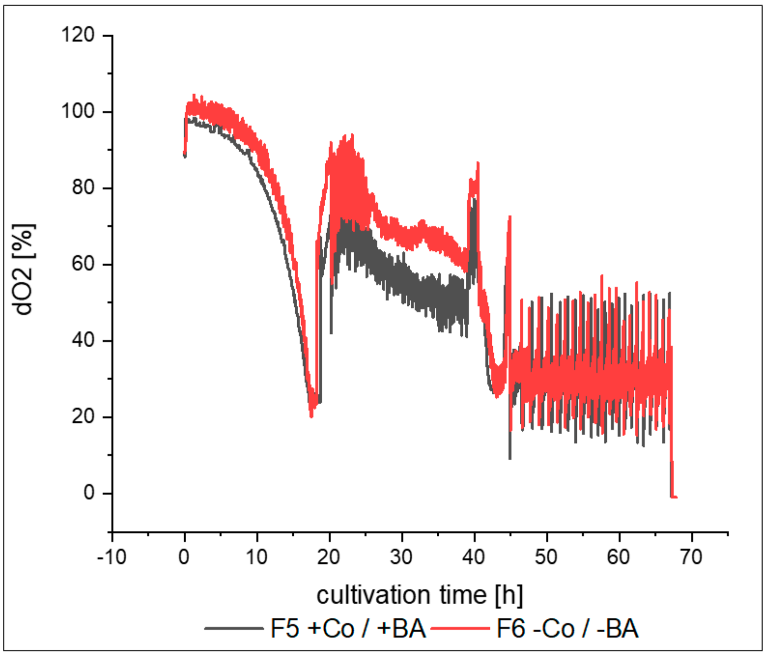

Figure S3. Process diagram of dO_2 from cultivations F5 and F6 for HRP production with and without cobalt (Co) and boric acid (BA). Batch phase is shown until ~20 h, and then fed-batch on glycerol, peak ~40 h indicates that feed was stopped. Then, methanol was pulsed and second peak ~43 h indicates metabolization of pulsed methanol and start of pulsed methanol feeding.

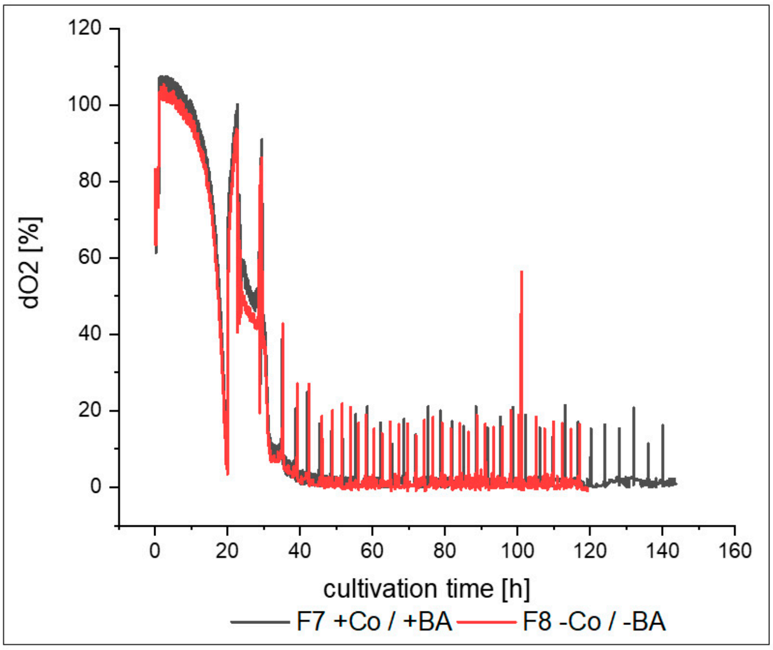

Figure S4. Process diagram of dO_2 from cultivations F7 and F8 for FC production with and without cobalt (Co) and boric acid (BA). Batch phase is shown until ~20 h, and then fed-batch on glycerol, peak ~30 h indicates that feed was stopped. Then, pulsed methanol feeding.

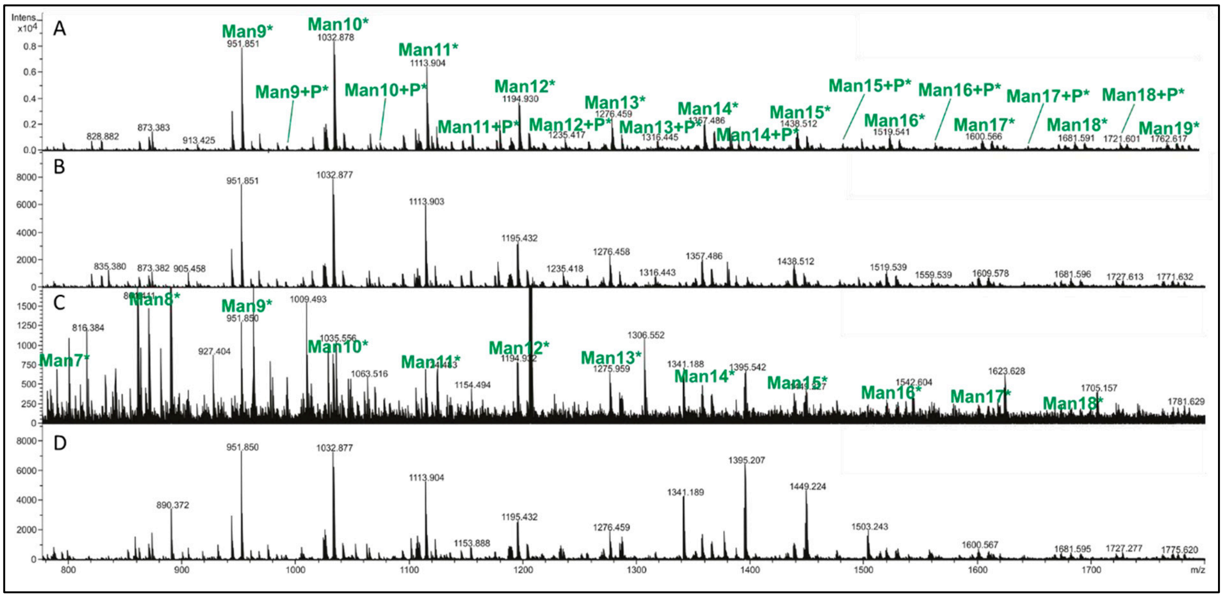

Figure S5. Results of LC-ESI-MS of PNGase F released N-glycans of purified HRP. The major glycoforms are high mannose type (Man8, Man9, Man9+Hex). Peaks with (*) indicate ammonium adducts. (**A**) HRP from F6 (**B**) HRP from F5 (**C**) HRP from F9 (**D**) HRP from F10. Glycoforms up to 24 residues were found (not shown).

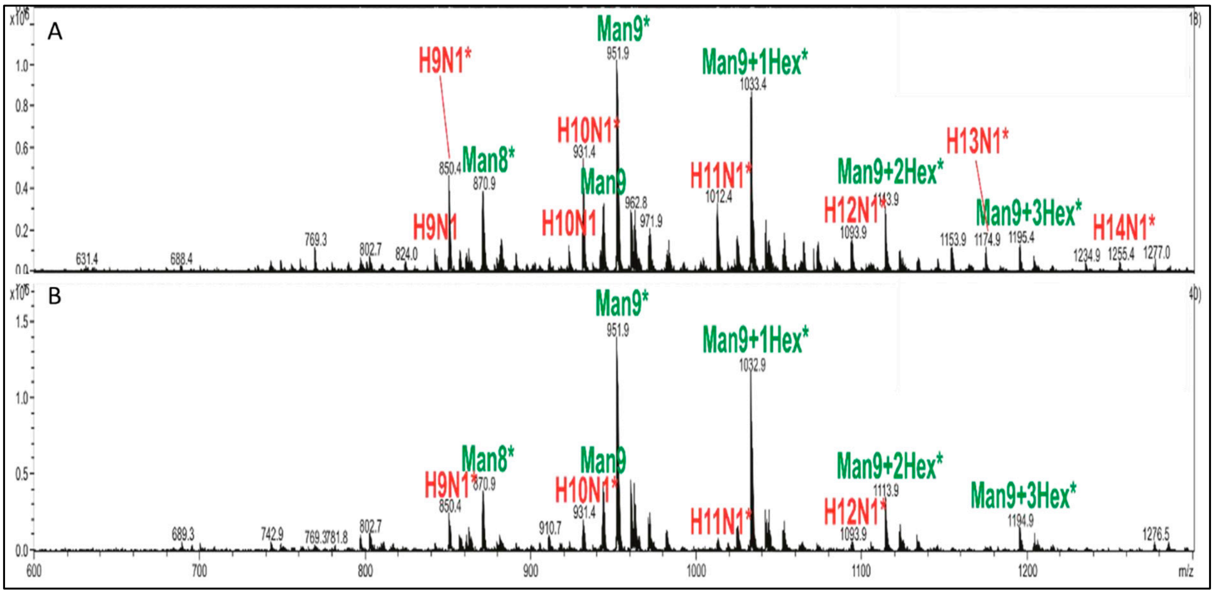

Figure S6. Results of LC-ESI-MS of PNGase F released N-glycans of purified FC. The major glycoforms are high mannose type (Man8, Man9, Man9+Hex). Peaks with lacking HexNAc were detected (H9N1 – H14N1). Peaks with (*) indicate ammonium adducts. (**A**) Purified FC of cobalt and boron salt supplemented cultivation C1. (**B**) Purified FC of cobalt and boron salt depleted cultivation C2.

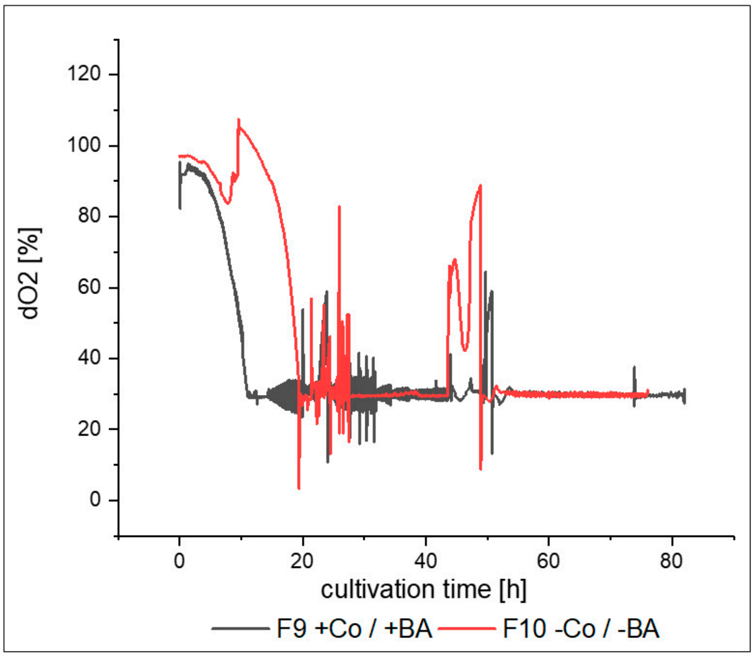

Figure S7. Process diagram of dO_2 from cultivations F9 and F10 for HRP production with and without cobalt (Co) and boric acid (BA). Batch phase is shown until ~24 h, then fed-batch on glycerol, peak around 43 h indicates that feed was stopped. Then, methanol was pulsed and dO_2 increase ~50 h indicates metabolization of pulsed methanol and start of exponential methanol feeding. The dO_2 control was set too sensitive in cultivation F9, which resulted in low visualization potential of the response to the methanol pulse.
